# Supplementary material for: Obesity-induced overexpression of miR-802 impairs insulin transcription and secretion
Source: Nat Commun. 2020 Apr 14;11:1822. doi: 10.1038/s41467-020-15529-w (PMC7156651; doi:10.1038/s41467-020-15529-w)
Supplement: Supplementary file 1 — Supplementary Information [file 41467_2020_15529_MOESM1_ESM.pdf]

Supplementary Information

Obesity-induced overexpression of *miR-802* impairs insulin transcription and secretion

Fangfang Zhang et al.

Supplementary Figure 1

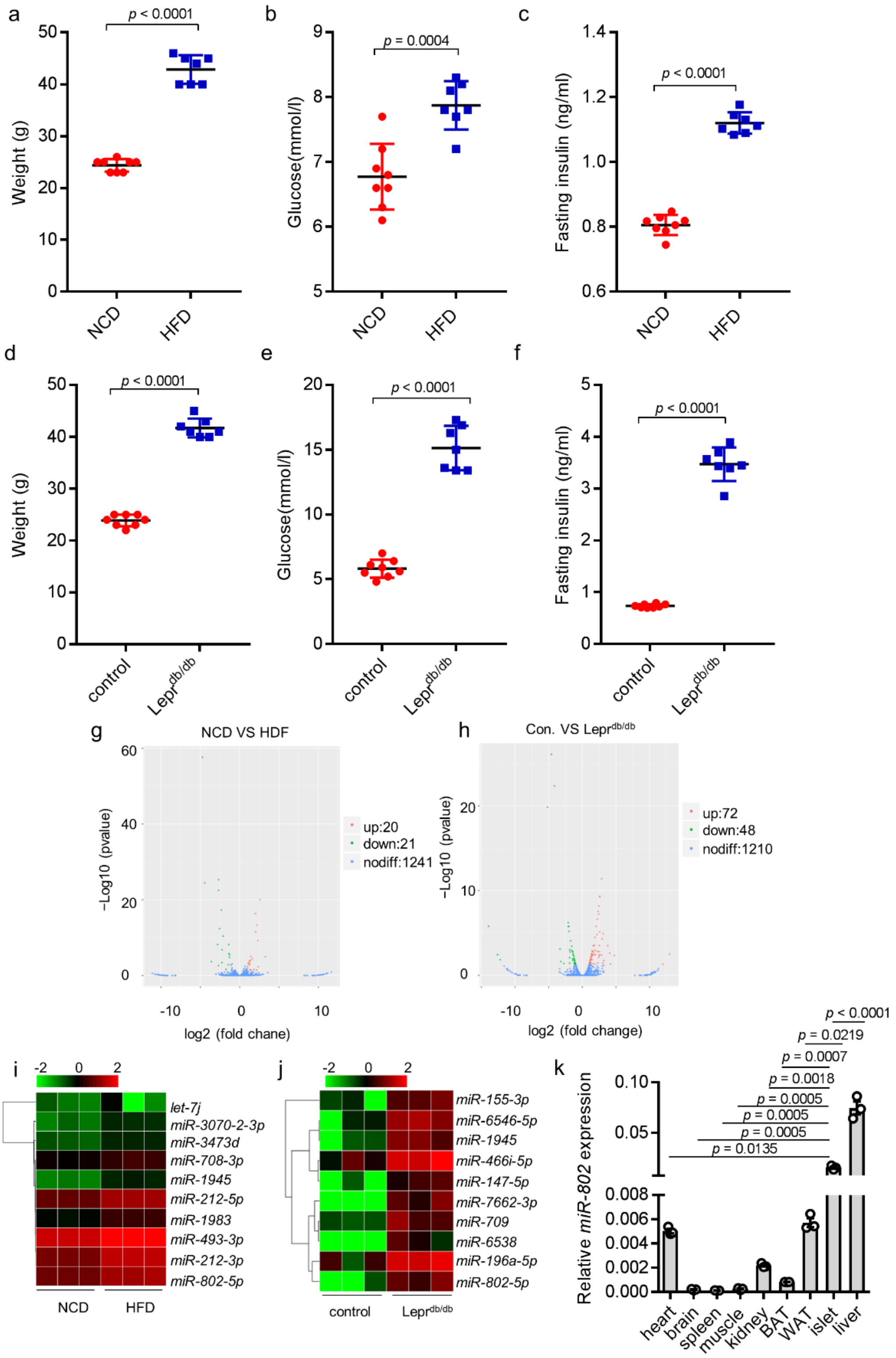

Supplementary Figure 1 The body weight, glucose and insulin level of HFD mice compared with NCD mice (a-c, *n* = 8 NCD group and *n* = 7 HFD group, 14-15-week old) and Lepr$^{db/db}$ mice compared with control mice (d-f, *n* = 8 control group and *n* = 7 Lepr$^{db/db}$ group, 8-week old). The volcano of miRNA in HFD (g, red bar: up, green bar: down and blue bar: no difference) versus

NCD mice, and Lepr$^{db/db}$ mice (h) versus Lepr$^{db/-}$ mice. Heat map diagram illustrating the expression levels of top 10 up-regulated miRNAs in islets of HFD mice (i), and in Lepr$^{db/db}$ mice (j) (n = 8 NCD group and n = 7 HFD group, n = 8 control group and n = 7 Lepr$^{db/db}$ group,). (k) The abundance of *miR-802* in different tissues of wide type mice by qRT-PCR analysis (n = 5, 8 weeks old). The p-values by two-tailed unpaired Student's t test a-f or one-way ANOVA k are indicated. All the results above were represented as mean ± SD, except a-f (mean ± SEM). Source data are provided as a Source Data file.

Supplementary Figure 2

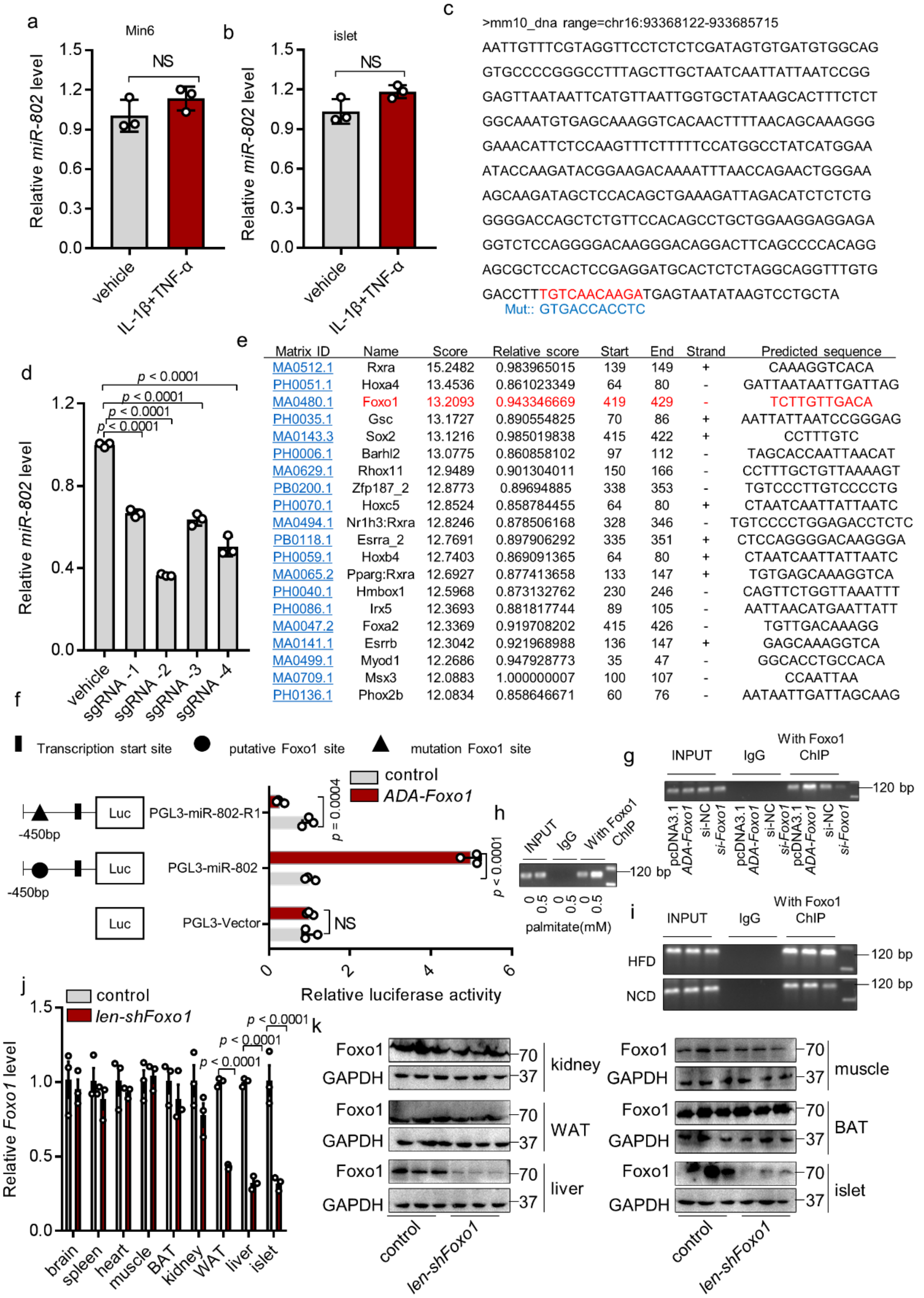

Matrix ID	Name	Score	Relative score	Start	End	Strand	Predicted sequence
MA0512.1	Rxra	15.2482	0.983965015	139	149	+	CAAAGGTCACA
PH0051.1	Hoxa4	13.4536	0.861023349	64	80	-	GATTAATAATTGATTAG
MA0480.1	Foxo1	13.2093	0.943346669	419	429	-	TCTTGTTGACA
PH0035.1	Gsc	13.1727	0.890554825	70	86	+	AATTATTAATCCGGGAG
MA0143.3	Sox2	13.1216	0.985019838	415	422	+	CCTTTGTC
PH0006.1	Barhl2	13.0775	0.860858102	97	112	-	TAGCACCAATTAACAT
MA0629.1	Rhox11	12.9489	0.901304011	150	166	-	CCTTTGCTGTTAAAAGT
PB0200.1	Zfp187_2	12.8773	0.89694885	338	353	-	TGTCCCTTGTCCCCTG
PH0070.1	Hoxc5	12.8524	0.858784455	64	80	+	CTAATCAATTATTAATC
MA0494.1	Nr1h3:Rxra	12.8246	0.878506168	328	346	-	TGTCCCCTGGAGACCTCTC
PB0118.1	Esrra_2	12.7691	0.897906292	335	351	+	CTCCAGGGGACAAGGGA
PH0059.1	Hoxb4	12.7403	0.869091365	64	80	+	CTAATCAATTATTAATC
MA0065.2	Pparg:Rxra	12.6927	0.877413658	133	147	+	TGTGAGCAAAGGTCA
PH0040.1	Hmbox1	12.5968	0.873132762	230	246	-	CAGTTCTGGTTAAATTT
PH0086.1	Irx5	12.3693	0.881817744	89	105	-	AATTAACATGAATTATT
MA0047.2	Foxa2	12.3369	0.919708202	415	426	-	TGTTGACAAAGG
MA0141.1	Esrrb	12.3042	0.921968988	136	147	+	GAGCAAAGGTCA
MA0499.1	Myod1	12.2686	0.947928773	35	47	-	GGCACCTGCCACA
MA0709.1	Msx3	12.0883	1.000000007	100	107	-	CCAATTAA
PH0136.1	Phox2b	12.0834	0.858646671	60	76	-	AATAATTGATTAGCAAG

Supplementary Figure 2 (a) Min6 cells and (b) primary islets were exposed to a combination of interleukin-1β (IL-1β, 5 ng mL^{-1}) and tumor necrosis factor-α (TNF-α, 30 ng mL^{-1}, black bar and white bar: vehicle) for 48 h. (c) The sequence of *miR-802* promoter; the red marker represents the binding site of *Foxo1* and blue marker represents the mutation sequences of binding site. (d) Four sgRNAs for predicted *miR-802* promoter region were constructed in lentiCRISPRv2 puro vector,

qRT-PCR was performed to determine the *miR-802* promoter region. (e) The high score transcription factor of *miR-802* promoter predicted by Jaspar and Promo. (f) A *Foxo1* binding site were identified in the -3 kb upstream region of the *miR-802* primary transcript. Mutagenesis in the putative binding site abrogated the induction activity of *Foxo1* in the Min6 cells. ChIP experiment showed that *Foxo1* binds to *miR-802* promoter via RT-PCR analysis, in the Min6 cells (g), in 0.5 mM palmitate-treated Min6 cells (h), and in obese mice islets (i, n = 5). (j-k) The Knockdown efficiency of Foxo1 were analyzed by qRT-PCR (j) and western blot (k, n = 5). All experiments above were performed in triplicates, where each group consisted of three samples. The p-values by two-tailed unpaired Student's t test a, b, one-way ANOVA d or two-way ANOVA f and j are indicated. NS, Not Significant. Data represent the mean ± SD. Source data are provided as a Source Data file.

Supplementary Figure 3

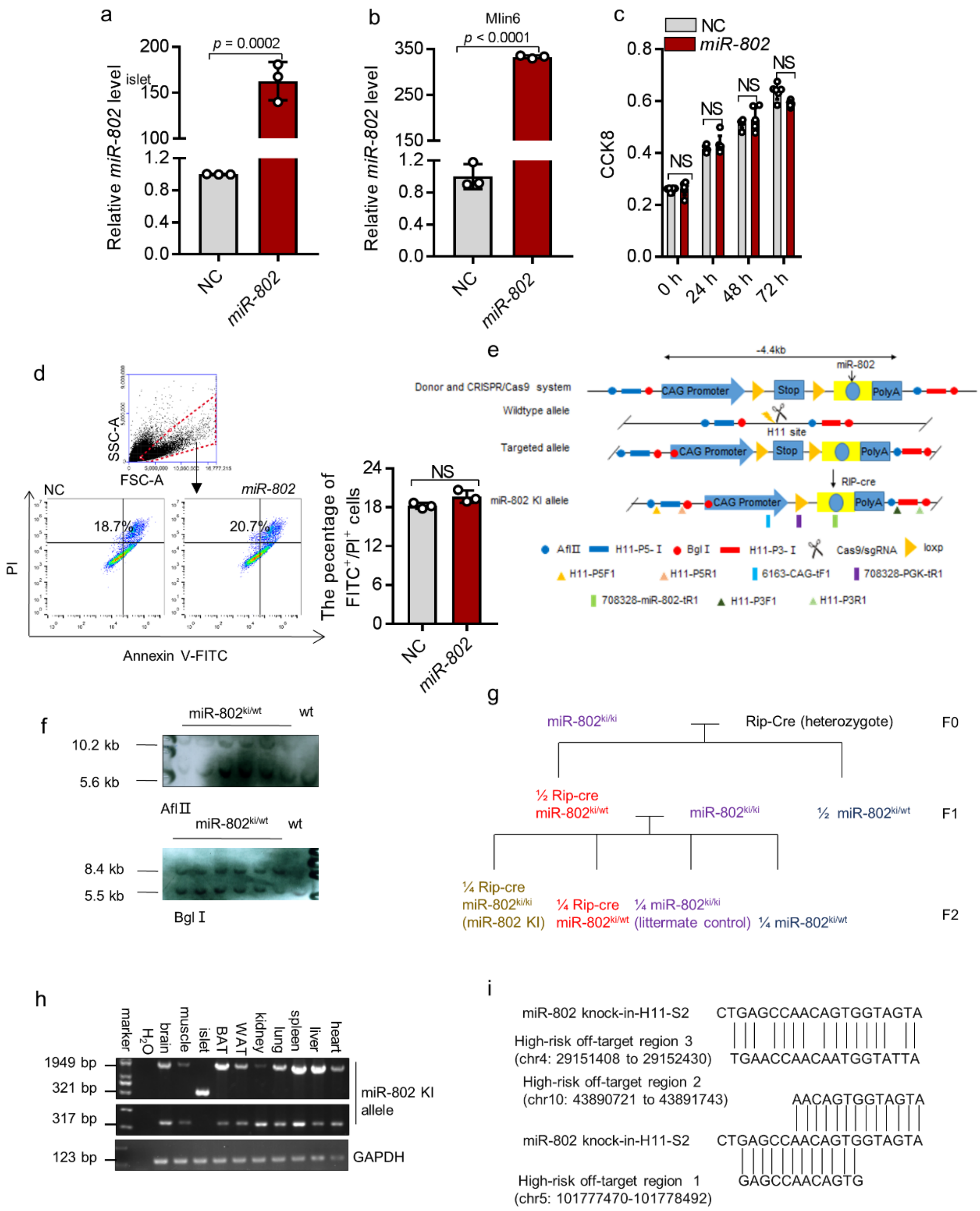

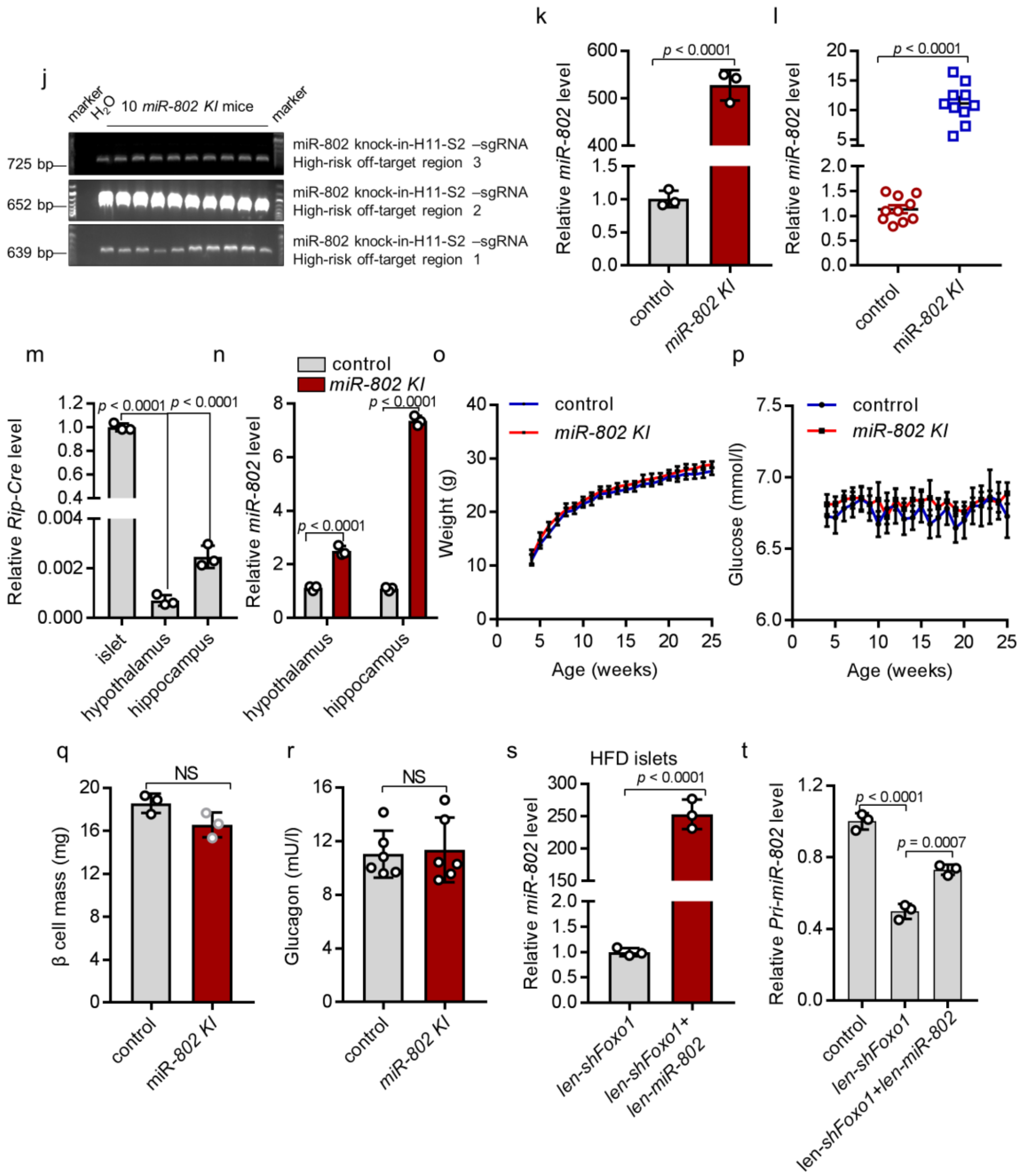

Supplementary Figure 3 The transfection efficiencies of *miR-802* (a) in islets (n = 3, 8 weeks old) and (b) in Min6 cells (n = 3 cells). (c) CCK-8 assay was performed at 24, 48, and 72 h (n = 6 cells), and flow cytometry was conducted 48 h after transfection, the numbers represent the percentage of $FITC^+$ cells and PI^+ cells. (d). (e) Strategy used to generate *miR-802* knock-in mice. Primers (H11-P5F1, H11-P5R1, H11-P3F1 and H11-P3R1) were used for southern blotting analyze. Primers (6163-CAG-tF1, 708328-PGK-tR1, 708328-miR802-tR1) were used for selective overexpression of the *miR-802* gene. (f) Southern blotting of tail genomic DNA (n = 6). Wild type allele: 5.6 kb for AflII and 5.5 kb for BglI; heterozygote targeted allele: 10.2 kb for AflII and 8.4 kb for BglI. (g) The cross flowchart to obtain *miR-802 KI* mice according to Mendelian inheritance. (h) Selective overexpression of the *miR-802* gene in islets (n = 5). Wild type allele revealed two bands, 317 bp and 1921 bp; mutant allele revealed only one band, 321 bp. (i) The high-risk off-target sequence of *miR-802* knock-in-H11-S2-sgRNA by [http://crispr.tefor.net/], (j) the off target effects (n = 10). (k)

miR-802 levels in primary islets (n = 5), in the serum (l, n = 10). (m) Rip-Cre expression in islet, hypothalamus and hippocampus from Rip-Cre mice (n = 5). *miR-802* expression in hypothalamus and hippocampus (n, n = 5), Body weight (o) and Adlibitum–fed blood glucose levels (p, n = 11 control group, n = 9 *miR-802 KI* group). (q) β cell mass (n = 3), (r) The glucagon levels (n = 6). (s) Overexpression efficiency of *miR-802* and *Pri-miR-802* levels (t, n = 5, 17-18 weeks old). All experiments above were performed in triplicates, where each group consisted of three samples. The p-values by two-tailed unpaired Student's t test a, b, d, k, l, q, r and s, one-way ANOVA m, and t, or two-way ANOVA c, m and n are indicated. All the results above were represented as mean ± SD except o-p (mean ± SEM). Source data are provided as a Source Data file.

Supplementary Figure 4

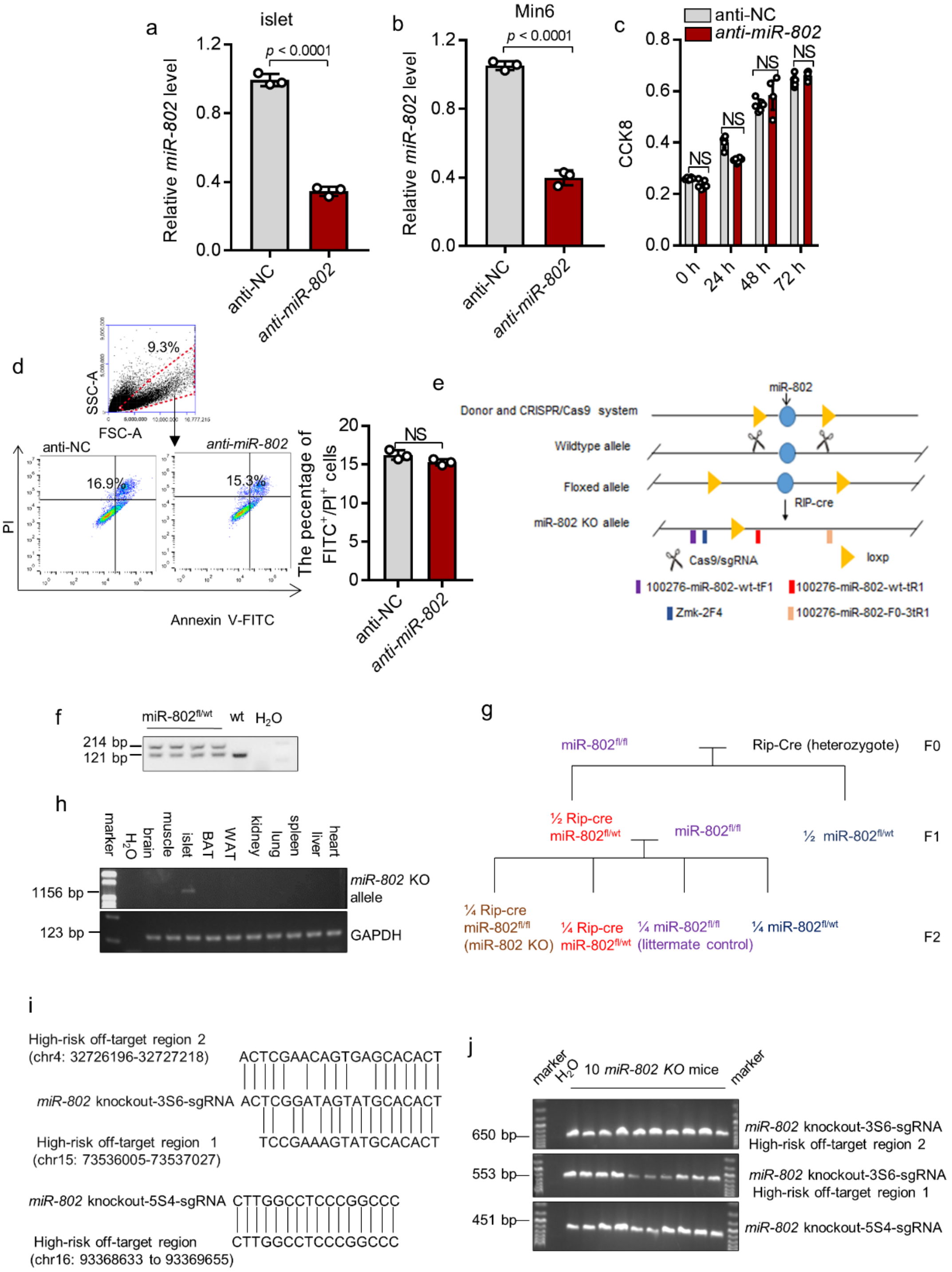

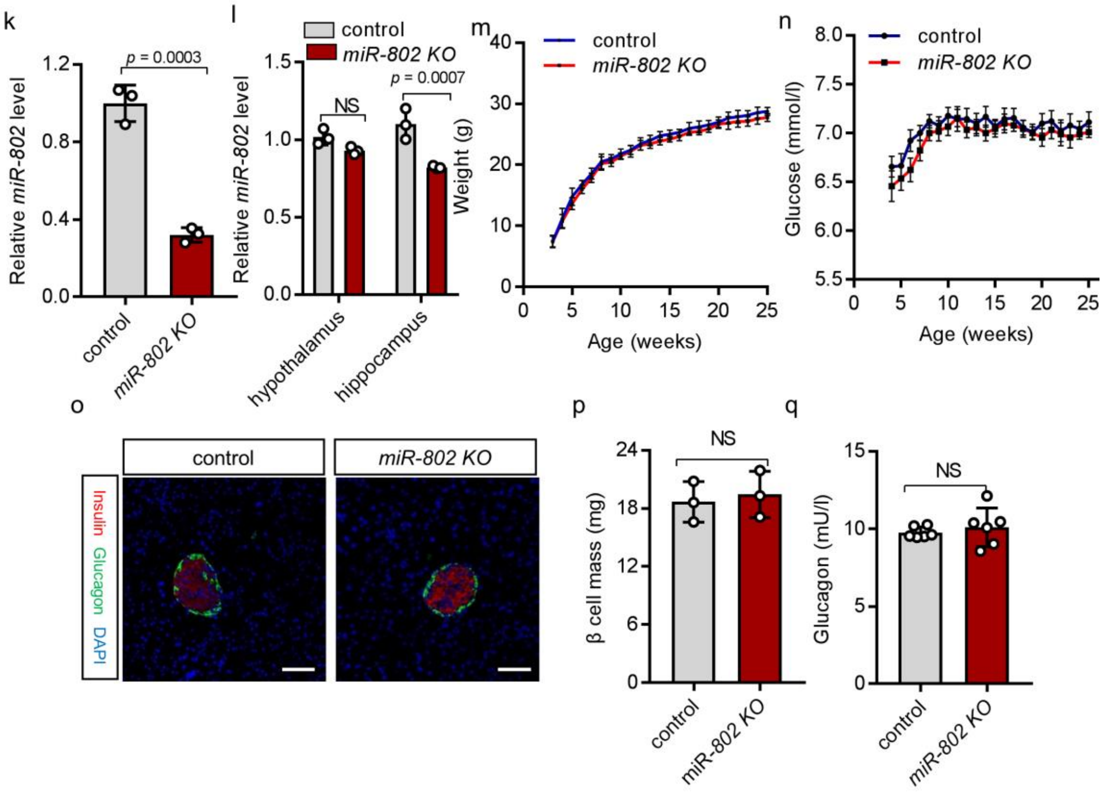

Supplementary Figure 4 The knockdown efficiencies of *miR-802* (a) in islets (n = 3, 8 weeks old) and (b) in Min6 cells. (c) CCK-8 assay was performed at 24, 48, and 72 h, and flow cytometry was conducted 48 h after transfection, the numbers represent the percentage of FITC$^+$ cells and PI$^+$ cells (d). (e) Strategy used to generate *miR-802* mutant mice. The primer of 100276-*miR-802*-wt-tF1 and 100276-*miR-802*-wt-tR1 were used to RT-PCR analyze. The primer of Zmk-2F4, and 100276-*miR-802*-F0-3tR1 were used to selective deletion of the *miR-802* gene in islets of *miR-802 KO* mice. (f) RT-PCR of tail genomic DNA isolated from wild type (wt) and heterozygote (*miR-802*$^{fl/wt}$, n = 4). *miR-802* mutant mice crossed with Deleter cre mice. Wild type allele revealed one bands, 121 bp; heterozygote allele revealed two band, 214 bp and 121 bp. (g) The cross flowchart to obtain *miR-802 KO* mice according to Mendelian inheritance. (h) Selective deletion of the *miR-802* gene in islets of *miR-802 KO* mice (n = 5). Genomic DNA from indicated mouse tissues was used in PCR with *miR-802* primers spanning LoxP sites (Top) and GAPDH as control (Bottom), white adipose tissue (WAT), and brown adipose tissue (BAT). (i) The high-risk off-target sequence of *miR-802* knockout-5S4-sgRNA and *miR-802* knockout-3S6-sgRNA via [http://crispr.tefor.net/], (j) the off target effects of *miR-802 KO* mice (n = 10). (k) Relative *miR-802* expression in islets of *miR-802 KO* and control mice (n = 5, 8 weeks old). (l) Relative *miR-802* expression level in hypothalamus and hippocampus of *miR-802 KO* mice (n = 5). (m) Body weight and Adlibitum–fed blood glucose levels (n, n = 9). (o) Islet organization (n = 3, 10 weeks old). Scale bar: 50 µm. (p) Pancreatic β cell mass (n = 3, 25-week old). (q) Glucagon levels (n = 6). All experiments above were performed in triplicates, where each group consisted of three samples. The p-values by two-tailed unpaired Student's t test a, b, d, k, p, and q, or two-way ANOVA c and l are indicated. Data represent the mean ± SEM. Source data are provided as a Source Data file.

Supplementary Figure 5

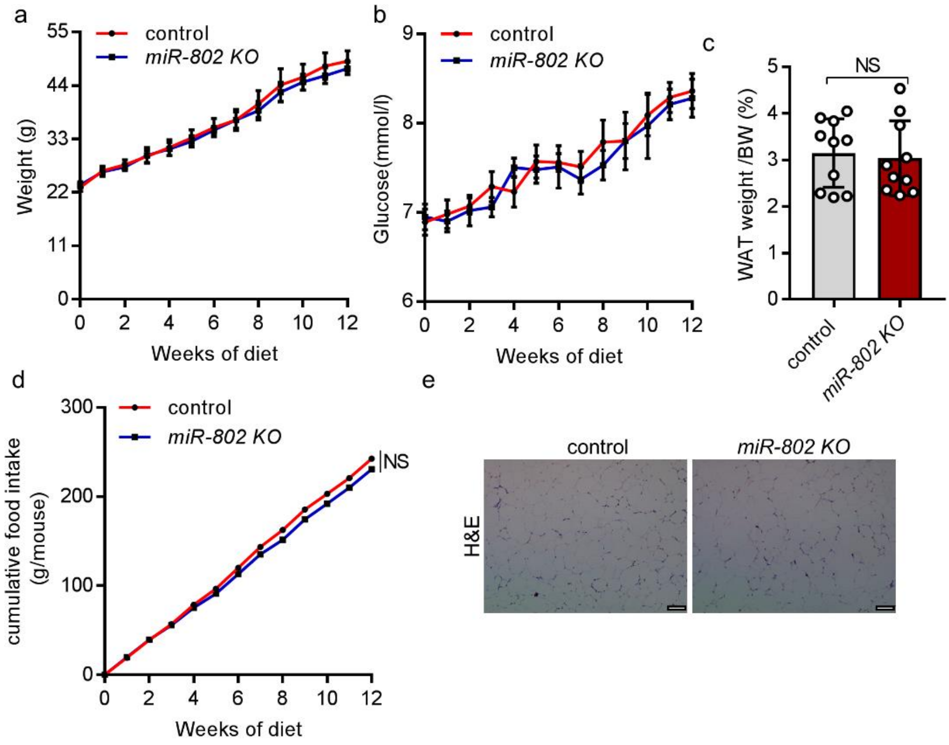

Supplementary Figure 5 (a) Changes in the body weight of *miR-802 KO* and control mice treated with a HFD for 12 weeks (*n* = 10 per time point), Fasting blood glucose levels (FBG) (b, *n* = 10), cumulative energy intake (c, *n* = 10), white adipose tissue weight per body weight ratio (d, *n* = 10) and representative H&E staining of white adipose tissue in *miR-802 KO* mice and control mice treated with HFD (e, Magnification: 4 ×, Scale bar: 100 µm, *n* = 5). FBG levels and cumulative energy intake were measured every 1 week. The *p*-values by two-tailed unpaired Student's t test c, or two-way ANOVA d are indicated. Data represent the mean ± SEM. Source data are provided as a Source Data file.

Supplementary Figure 6

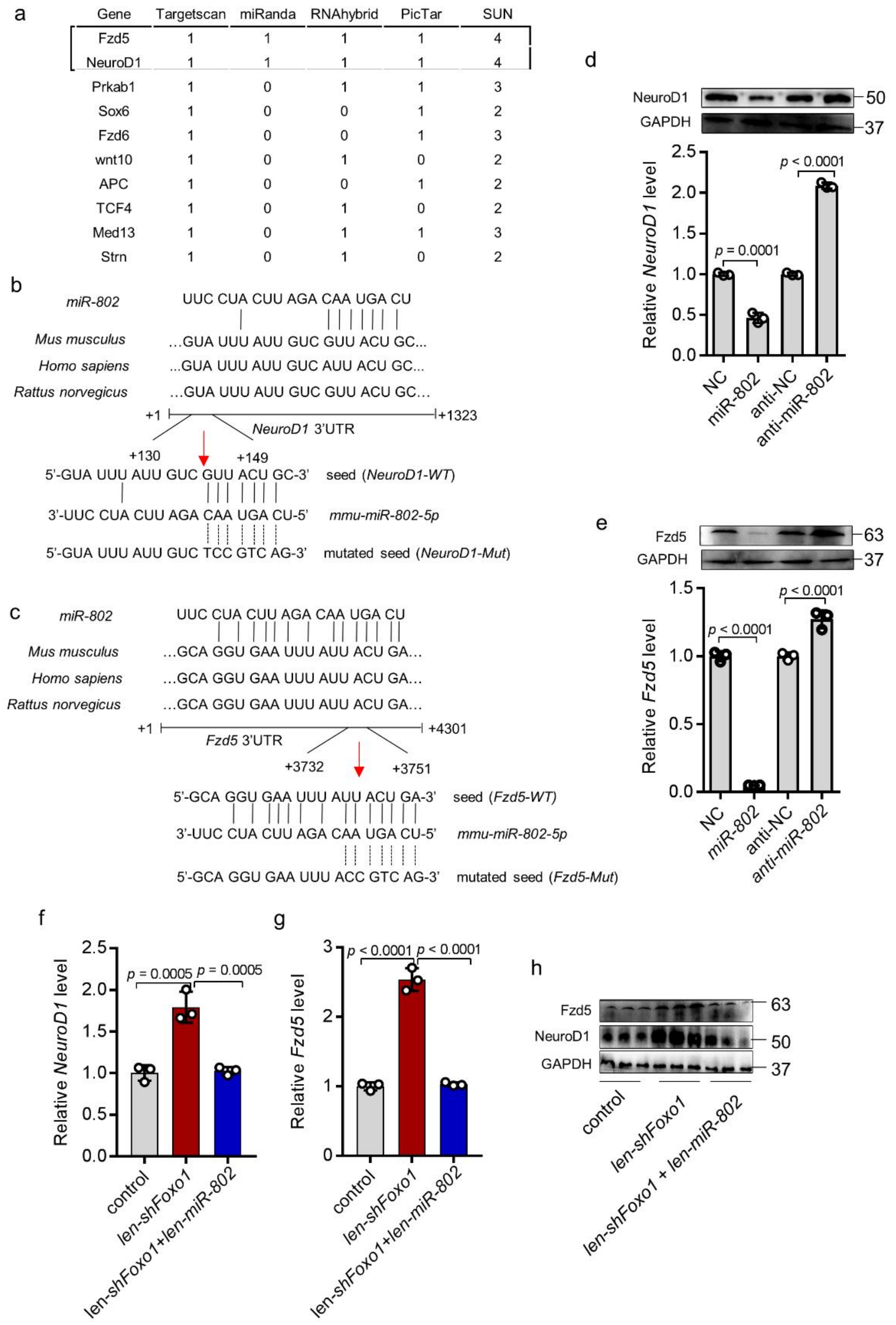

Supplementary Figure 6 (a) List of ten putative *miR-802* target genes with a seed sequence homology of > 6 nucleotides and simultaneously predicted by four independent miRNA target prediction algorithms. (b-c) Graphic representation of the conserved *miR-802* binding motif in the *NeuroD1* and *Fzd5* 3'UTR of three mammalian species. The consensus mature *miR-802* sequence was depicted on top. And schematic description of the wildtype (top)

and mutated (bottom) *miR-802* seed binding motif located in the murine *NeuroD1*- and *Fzd5*-3'UTR used for transient reporter gene transfection experiments. The mRNA and protein levels of NeuroD1 (d) and Fzd5 (e) in Min6 cells. The mRNA (f-g) and protein (h) levels of NeuroD1 and Fzd5 in the islet of HFD-fed mice injected with *lentivirus-shFoxo1 and lentivirus-miR-802*(*n* = 5, 17-18 weeks old). All experiments above were performed in triplicates, where each group consisted of three samples. The *p*-values by one-way ANOVA d, e, f and g are indicated. Data represent the mean ± SD. Source data are provided as a Source Data file.

Supplementary Figure 7

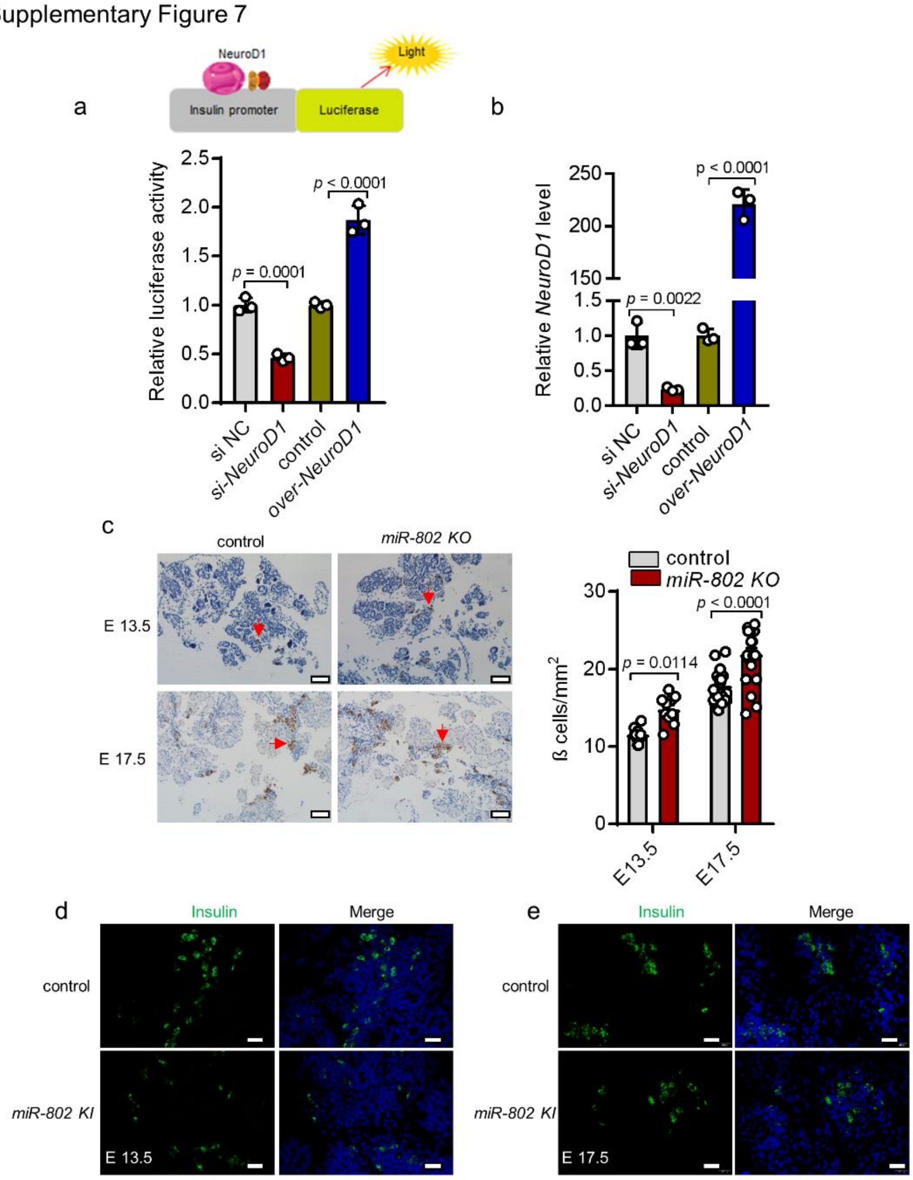

Supplementary Figure 7 (a) *NeuroD1* bound to insulin promoter as determined by dual luciferase assay. (b) Overexpression and knockdown *NeuroD1* in Min6 cells for 48 h; qRT-PCR was performed to evaluate the transfection efficiency. All experiments above were performed in triplicates, where each group consisted of three groups. All the results above were represented as mean ± SD, $**p < 0.01$, $***p < 0.001$ compared with the siNC or control. (c) Pancreatic sections of control mice and

miR-802 KO mice were immunohistochemically stained for insulin at E13.5 (upper) and E17.5 (lower). Scale bars: 50 µm (*n* = 3 mice 6–8 slides animal^{-1}). The number refers to insulin cell numbers/pancreas area. (d-e) Immunofluorescence of insulin (green) and DAPI (blue) in E13.5 (d) and E17.5 (e) pancreas of control mice and *miR-802 KI* mice (*n* = 3 mice). Data information: The *p*-values by two-tailed unpaired Student's t test b, one-way ANOVA a, or two-way ANOVA c are indicated. Data are presented as means ± SD. Source data are provided as a Source Data file. Scale bars: 50 µm (c); 20 µm (d, e).

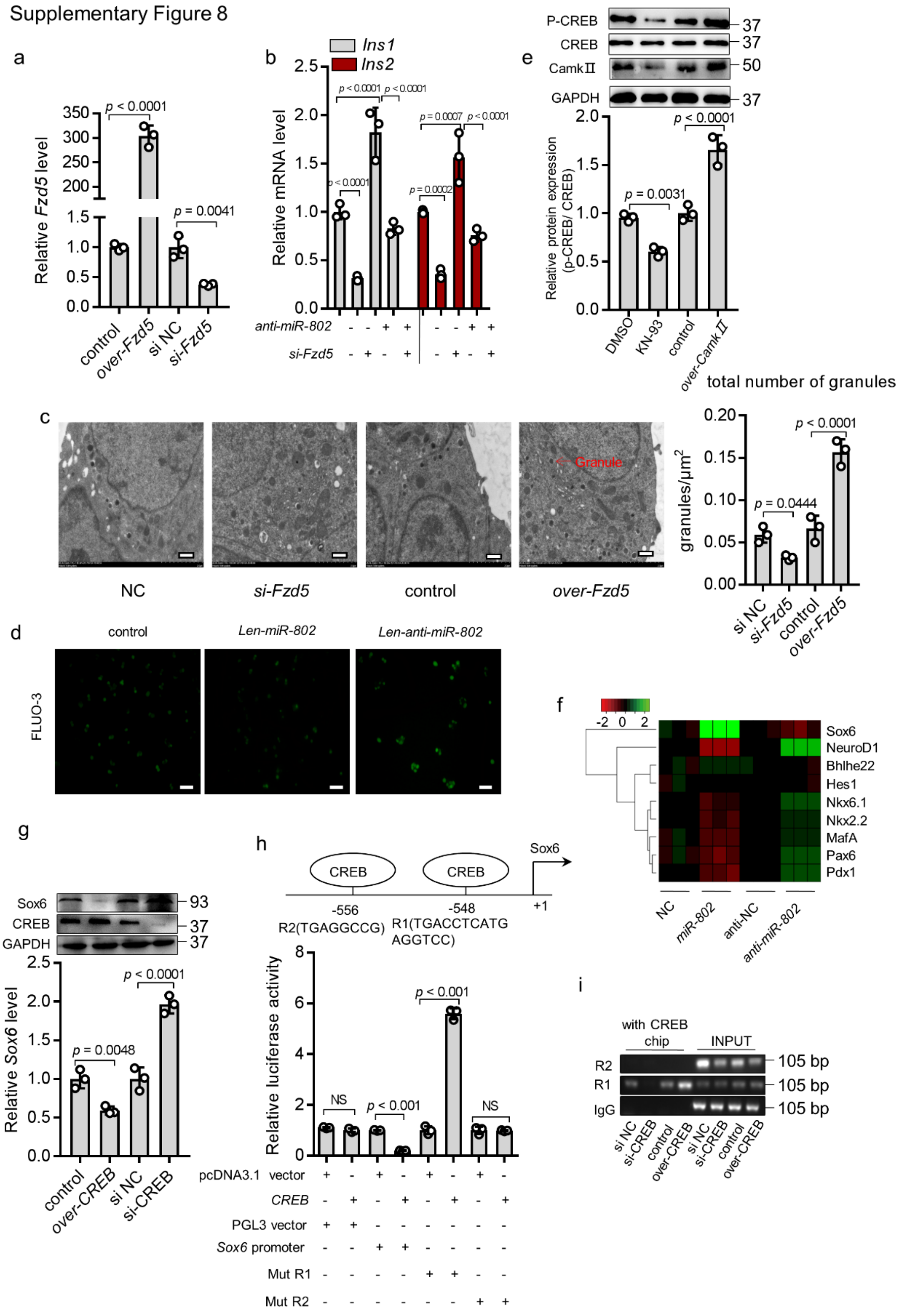

Supplementary Figure 8 (a) The transfection efficiency of *Fzd5* was analyzed by qRT-PCR when *over-Fzd5* vector or *si-Fzd5* were transfected in Min6 cells for 48 h. (b) Min6 cells were co-transfected with si NC or *si-Fzd5* and anti-NC or *anti-miR-802*. After 48 h, *Ins1* and *Ins2* expression levels were evaluated by qRT-PCR. (c) Overexpression and knockdown of *Fzd5* in Min6 cells; granules were quantified by electron microscopy. Scale bars: 20 μm. (d) Overexpression and

knockdown of *miR-802* in Min6 cells for 48 h; intracellular Ca^{2+} content was measured by FLUO-3. Scale bars: 50 µm. (e) Western blot was performed to analyze the phosphorylation level of CREB when *CamkII* was overexpressed or suppressed. (f) Transfected *miR-802* mimics and *anti-miR-802* in Min6 cells; qRT-PCR was conducted to evaluate the islet transcription factors. (g) *si-CREB* or *CREB* was transfected into Min6 cells. qRT-PCR and western blot were conducted to examine the expression level of Sox6. (h) Two *CREB* binding sites were identified in the -2 kb upstream region of the *Sox6* promoter (top), mutagenesis in the putative binding site of *CREB* enhanced the induction of *Sox6* in the Min6 cells (bottom). (i) ChIP experiment showed that *CREB* binds to the promoter of *Sox6* by RT-PCR assays. Only binding site R1 showed positive results. All experiments above were performed in triplicates, where each group consisted of three samples. The *p*-values by one-way ANOVA a, e, c, g and h, or two-way ANOVA b are indicated. Data represent the mean ± SD. Source data are provided as a Source Data file.

The sequence of Rat insulin2 promoter (704 bp)

GGATCCCCCAACCACTCCAAGTGGAGGCTGAGAAAGGTTTTGTAGCTGGGTAGAGTAT
GTACTAAGAGATGGAGACAGCTGGCTCTGAGCTCTGAAGCAAGCACCTCTTATGGAGA
GTTGCTGACCTTCAGGTGCAAATCTAAGATACTACAGGAGAATACACCATGGGCTTCA
GCCCAGTTGACTCCCGAGTGGGCTATGGGTTTGTGGAAGGAGAGATAGAAGAGAAGG
GACCTTTCTTCTTGAATTCTGCTTTCCTTCTACCTCTGAGGGTGAGCTGGGGTCTCAGC
TGAGGTGAGGACACAGCTATCAGTGGGAACTGTGAAACAACAGTTCAAGGGACAAAG
TTACTAGGTCCCCCAACAACTGCAGCCTCCTGGGGAATGATGTGGAAAAATGCTCAGC
CAAGGACAAAGAAGGCCTCACCCTCTCTGAGACAATGTCCCCTGCTGTGAACTGGTTC
ATCAGGCCACCCAGGAGCCCCTCTTAAGACTCTAATTACCCTAAGGCTAAGTAGAGGT
GTTGTTGTCCAATGAGCACTTTCTGCAGACCTAGCACCAGGCAAGTGTTTGGAAACTG
CAGCTTCAGCCCCTCTGGCCATCTGCTGATCCACCCTTAATGGGACAAACAGCAAAGT
CCAGGGGTCAGGGGGGGGGTGCTTTGGACTATAAAGCTAGTGGGGATTCAGTAACCCC
CAGCCCTAA

Supplementary Table 1 The sgRNA sequences of miR-802 knockout and knock-in mice

sgRNA name	sgRNA sequence(5’-3’)	PAM
miR-802 knockout-5S4	TGAGCTTGGCCTCCCGGCCC	TGG
miR-802 knockout-3S6	ACTCGGATAGTATGCACACT	CGG
miR-802 knock-in-H11-S2	CTGAGCCAACAGTGGTAGTA	AGG

Supplementary Table 2 Primer sequences used genotyping of miR-802 mutant mice

name	primer name	sequence	Band (bp)
Rip-Cre Genotyping	oIMR1084	GCGGTCTGGCAGTAAAAA CTATC	Cre = ~100
	oIMR1085	GTGAAACAGCATTGCTGTCAC TT	
	oIMR7338	CTA GGC CAC AGA ATT GAA AGA TCT	Internal Positive Control=324
	oIMR7339	GTA GGT GGA AAT TCT AGC ATC ATC C	
miR-802$^{fl/fl}$ Genotyping	100276-miR-802-wt-tF1	TGCCGAGAATGATTGCTGCA	wt=121<br>fl=214
	100276-miR-802-wt-tR1	TGTTACCGACATTCCCCTTAGA	
miR-802 KO allele	Zmk-2F4	GCATCGCATTGTCTGAGTAGGTG	null=1156
	100276-miR-802-F0-3tR1	AGTGCAGTTACCCGTCACCA	
miR-802$^{ki/ki}$ Genotyping	Rabbit-pA-tF1	CCTGCTGTCCATTCCTTATTCCATA	ki/ki=337<br>ki/wt=337<br>wt/wt=none
	H11-tR3	ATATCCCCTTGTTCCCTTTCTGC	
	H11-tF3	GGGCAGTCTGGTACTTCCAAGCT	ki/ki=none<br>ki/wt=285<br>wt/wt=285
	H11-tR3	ATATCCCCTTGTTCCCTTTCTGC	
miR-802 KI allele	6163-CAG-tF1	CTAGAGCCTCTGCTAACCATGTTC	ki(preCre)=317<br>ki(postCre)=0<br>wt=0
	708328-PGK-tR1	AAGAGCCTTCAGTAAAGAGCAGG	
	6163-CAG-tF1	CTAGAGCCTCTGCTAACCATGTTC	ki(preCre)=1949<br>ki(postCre)=321
	708328-miR802-tR1	AGAGCAGGCATTATCTTGCCAC	
High-risk off-target of miR-802 knockout-5S4	XM100276-miR-802-5S4-tF1	TTATTGTGTGACTCCAGGGCTG	451
	XM100276-miR802-5S4-tR1	GTGTCAAATCCCAGCCTGATG	
High-risk off-target of miR-802 knockout-3S6 region 1	XM100276-miR-802-3S6-tF1	CCAAGTATGCCTTGTCTTTCAGC	553
	XM100276-miR-802-3S6-tR1	CCATGTTGGCCTAGTCAGACTTG	
High-risk off-target of miR-802 knockout-3S6 region 2	XM100276-miR-802-3S6-tF2	ACAGCATTTAGCTGAGCACCTG	650
	XM100276-miR-802-3S6-tR2	TGCTCACTAATGCAGTGACAGC	
High-risk off-target of miR-802 knock-in-H11-S2 region 1	XM708328-H11-S2-tF1	GATGGCTCGCAAACATCTTTG	639
	XM708328-H11-S2-tR1	CTGTGGCTTTGTAAAGTTGTCTG G	
High-risk off-target of miR-802 knock-in-H11-S2 region 2	XM708328-H11-S2-tF2	CTGGAGTGATTATGAGATGCCTG	652
	XM708328-H11-S2-tR2	CTGACCTCCATCCTTTAGCAAC	
High-risk off-target of miR-802 knock-in-	XM708328-H11-S2-tF3	GATTCCATTTCTAGCTCTGCCAG	725 bp
	XM708328-H11-S2-tR3	GAACTGCTTTGCCTGCACTTAAC	

H11-S2 region 3			

Supplementary Table 3 Clinical characteristics of the patients with obese patients and lean individuals

	Obesity	Lean	Total
Number (male/female)	75 (44/31)	22(8/14)	97(52/45)
Age (years)	52.34±9.41	54.68±8.69	52.87±9.99
HbA1c (%)	7.73±2.14	5.67±0.47	7.28±2.08
TG (mmol/L)	1.55±1.11	0.91±0.43	1.41±1.04
HDL-C (mmol/L)	0.98±0.35	1.14±0.19	1.02±0.33
LDL-C (mmol/L)	2.46±0.92	2.29±0.50	2.42±0.85
BMI	30.73±2.7	22.07±1.87	28.53±4.87

Supplementary Table 4 List of miRNAs upregulated (top) and downregulated (bottom) in HFD-fed mice (p<0.05) Microarray analysis was performed in quadruplicate and mean ± standard error is indicated. The *p*-values by two-tailed unpaired Student's t test

miRNA	Log2(X-fold (HFD/NCD))	*p*-value
mmu-miR-1945	3.242035183	1.66E-07
mmu-miR-212-3p	2.556646996	3.84E-23
mmu-miR-3070-2-3p	2.361083788	0.000141
mmu-miR-1983	2.311880436	5.65E-12
mmu-miR-212-5p	2.183221843	3.13E-16
mmu-let-7j	2.146049745	0.001357
mmu-miR-802-5p	2.02295942	2.31E-14
mmu-miR-3473d	1.799682485	0.000823
mmu-miR-708-3p	1.401474069	1.35E-07
mmu-miR-493-3p	1.375974279	0.001105
mmu-miR-1a-3p	1.368004354	1.64E-06
mmu-miR-133b-3p	1.267212042	0.001017
mmu-miR-679-5p	1.255650988	1.48E-05
mmu-miR-144-3p	1.242031741	2.24E-06
mmu-miR-15b-3p	1.226077034	3.78E-05
mmu-miR-455-5p	1.172391009	5.64E-06
mmu-miR-708-5p	1.069166201	1.56E-05
mmu-miR-666-5p	1.008341378	0.000129
mmu-miR-672-5p	-1.006771527	0.001072
mmu-miR-5121	-1.043046822	0.001178
mmu-miR-150-5p	-1.297323405	2.44E-07
mmu-miR-142a-3p	-1.300325043	4.68E-08
mmu-miR-184-3p	-1.385620116	2.00E-08
mmu-miR-133-5p	-1.3931178	6.76E-11
mmu-miR-592-5p	-1.469661042	3.30E-05
mmu-miR-203-5p	-1.757017156	0.000737
mmu-miR-216b-3p	-2.115056759	3.70E-13
mmu-miR-216a-3p	-2.258472925	1.98E-09
mmu-miR-6969-5p	-2.269401306	8.13E-06
mmu-miR-216b-5p	-2.333897502	2.29E-20
mmu-miR-217-3p	-2.553989506	2.98E-15
mmu-miR-216a-5p	-2.679137841	8.02E-29
mmu-miR-217-5p	-2.693352297	9.63E-26
mmu-miR-1934-5p	-2.701028109	6.46E-05
mmu-miR-6969-3p	-2.819368527	7.07E-11
mmu-miR-196b-5p	-3.607161183	3.92E-06
mmu-miR-122-3p	-4.452340005	8.10E-28
mmu-miR-122-5p	-4.734692003	1.81E-61

Supplementary Table 5 List of miRNAs upregulated (top) and downregulated (bottom) in $Lepr^{db/db}$ mice (p<0.005) Microarray analysis was performed in quadruplicate and mean ± standard error is indicated. The *p*-values by two-tailed unpaired Student's t test

miR_name	X-fold ($Lepr^{db/db}$/con.)	*p*-value
mmu-miR-7622-3p	12.92840955	0.000104
mmu-miR-6538	11.94005362	0.004345
mmu-miR-802-5p	4.739058274	0.000211
mmu-miR-147-5p	4.146424818	0.003876
mmu-miR-6546-5p	3.753234851	5.73E-06
mmu-miR-709	3.172334817	0.001207
mmu-miR-196a-5p	3.079092249	1.63E-07
mmu-miR-155-3p	3.020048439	0.002683
mmu-miR-466i-5p	2.940448685	7.63E-07
mmu-miR-1945	2.84521787	0.000178
mmu-miR-212-3p	2.836469225	1.22E-14
mmu-miR-212-5p	2.562166853	1.79E-12
mmu-miR-196b-5p	2.55155411	9.82E-08
mmu-miR-712-5p	2.542128077	0.000148
mmu-miR-3470b	2.49695525	4.21E-05
mmu-miR-147-3p	2.495521522	6.02E-06
mmu-miR-3070-3p	2.48020014	0.00022
mmu-miR-155-5p	2.447619293	3.35E-11
mmu-miR-3070-2-3p	2.404508992	0.000396
mmu-miR-21a-3p	2.39899492	4.07E-10
mmu-miR-223-5p	2.329883871	0.002766
mmu-miR-150-5p	2.329498447	1.64E-10
mmu-miR-142a-3p	2.210419227	1.80E-09
mmu-miR-150-3p	2.210050735	3.91E-05
mmu-miR-1983	2.154430526	2.11E-07
mmu-miR-132-3p	2.034436499	1.96E-08
mmu-miR-6240	1.919699857	0.003968
mmu-miR-211-5p	1.909981728	0.000129
mmu-miR-215-5p	1.892510714	2.55E-05
mmu-miR-142a-5p	1.832136907	4.88E-07
mmu-miR-501-5p	1.763617281	5.13E-06
mmu-miR-214-5p	1.728146138	5.03E-06
mmu-miR-1247-5p	1.725033273	0.000145
mmu-miR-190b-5p	1.663537357	5.17E-05
mmu-miR-494-5p	1.62364173	0.003636
mmu-miR-708-5p	1.591629885	6.65E-06
mmu-miR-199b-5p	1.453012534	3.02E-05
mmu-miR-223-3p	1.443709818	4.67E-05

mmu-miR-503-3p	1.430467307	0.000895
mmu-miR-15b-3p	1.395624945	0.000197
mmu-miR-708-3p	1.378245396	9.09E-05
mmu-miR-20b-5p	1.373926743	0.000299
mmu-miR-145a-5p	1.373026407	0.000176
mmu-miR-199b-3p	1.369104842	0.000127
mmu-miR-199a-3p	1.368803458	0.000128
mmu-miR-3068-5p	1.350563922	0.000156
mmu-miR-455-3p	1.348598308	0.000197
mmu-miR-574-5p	1.338497327	0.000152
mmu-miR-450b-5p	1.312816779	0.002884
mmu-miR-451a	1.298044582	0.000699
mmu-miR-144-3p	1.292363281	0.000333
mmu-miR-214-3p	1.258818011	0.000358
mmu-miR-455-5p	1.24031328	0.00039
mmu-miR-22-3p	1.202332445	0.00109
mmu-miR-351-5p	1.199418314	0.000997
mmu-miR-199a-5p	1.194652843	0.000764
mmu-miR-139-5p	1.180552291	0.000677
mmu-miR-183-5p	1.155478377	0.000981
mmu-miR-133a-3p	1.148945779	0.001382
mmu-miR-99a-3p	1.147943073	0.004375
mmu-miR-671-5p	1.147755119	0.001142
mmu-miR-106a-5p	1.145428438	0.004257
mmu-miR-679-5p	1.130355708	0.00269
mmu-miR-143-3p	1.122403571	0.002155
mmu-miR-511-3p	1.096036208	0.003361
mmu-miR-322-5p	1.088724312	0.001824
mmu-miR-1a-3p	1.088618734	0.002065
mmu-miR-146a-5p	1.04898809	0.002652
mmu-miR-145a-3p	1.037717036	0.002884
mmu-miR-187-3p	-1.009591737	0.004028
mmu-miR-374b-5p	-1.016060002	0.004368
mmu-miR-872-3p	-1.022333925	0.003467
mmu-miR-153-3p	-1.062884819	0.003965
mmu-miR-411-5p	-1.095410417	0.002228
mmu-miR-3547-3p	-1.109112495	0.002976
mmu-miR-338-3p	-1.148638366	0.001267
mmu-miR-23b-3p	-1.159663732	0.001411
mmu-miR-210-3p	-1.16046995	0.001143
mmu-miR-26a-5p	-1.162788536	0.002722
mmu-miR-101a-3p	-1.188789747	0.001206

mmu-miR-341-3p	-1.199881681	0.000643
mmu-miR-31-5p	-1.204148408	0.000733
mmu-miR-384-5p	-1.210468608	0.000696
mmu-miR-33-3p	-1.232309772	0.002638
mmu-miR-652-5p	-1.235103796	0.003844
mmu-let-7e-5p	-1.245524128	0.000702
mmu-miR-384-3p	-1.293310747	0.000282
mmu-miR-153-5p	-1.31684577	0.000535
mmu-miR-700-3p	-1.35306301	0.000158
mmu-miR-204-5p	-1.368403349	0.000141
mmu-miR-124-3p	-1.426298494	0.00011
mmu-miR-325-3p	-1.429846754	4.98E-05
mmu-miR-217-5p	-1.434265352	5.73E-05
mmu-miR-216a-3p	-1.441308542	0.001185
mmu-miR-544-5p	-1.461554381	0.000662
mmu-miR-137-3p	-1.465802511	3.82E-05
mmu-miR-203-3p	-1.596590931	7.40E-06
mmu-miR-217-3p	-1.617809707	2.61E-05
mmu-miR-216a-5p	-1.756193658	1.37E-06
mmu-miR-383-3p	-1.83575558	5.13E-05
mmu-miR-592-5p	-1.901226007	2.16E-05
mmu-miR-7116-3p	-1.921791107	0.000432
mmu-miR-187-5p	-1.92272856	0.002908
mmu-miR-31-3p	-1.990991408	8.00E-08
mmu-miR-383-5p	-2.023849089	1.94E-08
mmu-miR-216b-3p	-2.116608137	1.72E-08
mmu-miR-216b-5p	-2.128540211	5.07E-09
mmu-miR-344i	-2.772201565	0.003301
mmu-miR-6971-3p	-3.244194026	0.001657
mmu-miR-5125	-3.34737383	1.77E-05
mmu-miR-122-5p	-4.181992387	6.60E-26
mmu-miR-184-3p	-4.613196989	6.27E-30
mmu-miR-122-3p	-5.201003989	3.19E-23
mmu-miR-7092-3p	-12.22941215	0.000868
mmu-miR-6897-5p	-12.59557723	0.000154
mmu-miR-3080-5p	-12.62911828	0.000153
mmu-miR-1934-5p	-13.95123036	1.43E-08

Supplementary Table 6 Primer sequences used for RT-PCR

Gene	Forward strand (5'-3')	Reverse strand (5'-3')
over-Fzd5	CGGGATCCCGATGGCTCGACCCGACCCGTCT	GCTCTAGAGCCTATACGTGCGACAGGGAC ACTTGC
over-CREB	CGGGATCCCGATGACCATGGAATCTGGAGCA G	CCCTCGAGGGTTAATCTGATTTGTGGCAG TAAAGG
over- CamkⅡ	CGGGATCCCGATGGCTACCATCACCTGCACC C	CCCTCGAGGGTCAATGCGGCAGGACGGA G
over-NeuroD1	CGGAATTCCGGCCACCATGACCAAATCATAC AGCGAGAG	CGGGATCCCCTAATCGTGAAAGATGGCAT TAAG
over-Foxo1	CGGGATCCCCGGCCACCATGGCCGAAGCGC CCCAGGTGGTG	CCCTCGAGTTAGCCTGACACCCAGCTGTG TG
over-Sox6	CCAAGCTTGGGCCACCATGTCTTCCAAGCAA GCCACCTCTC	CCCTCGAGTCAGTTGGCACTGACAGGCTC TGGG
Fzd5-WT	TCGAGACGGGTTGGTGCAGGTGAATTTATTA CTGAGCCAAATGAGGCACATACCGAGTT	CTAGAACTCGGTATGTGCCTCATTTGGCT CAGTAATAAATTCACCTGCACCAACCCGT C
Fzd5-mut	TCGAGACGGGTTGGTGCAGGTGAATTTACCG TCAGGCCAAATGAGGCACATACCGAGTT	CTAGAACTCGGTATGTGCCTCATTTGGCC TGACGGTAAATTCACCTGCACCAACCCGT C
NeuroD1-WT	TCGAGCAAGTGATATATGTATTTATTGTCGT TACTGCCTTTGGAAGAAACAGGGGATT	CTAGAGATCCCCTGTTTCTTCCAAAGGCA GTAACGACAATAAATACATATATCACTT C
NeuroD1-mut	TCGAGCAAGTGATATATGTATTTATTGTCAC CGTCACCTTTGGAAGAAACAGGGGATT	CTAGAGATCCCCTGTTTCTTCCAAAGGTG ACGGTGACAATAAATACATATATCACTT C
Sox6 R1	CAAGCCCAAGGTTTCGAGG	GAAGCCATTCTCCATCATTGTT
Sox6 R2	TGTGCTCACAAAGGGCTCATA	CTAGTCAAGCCATCATGCAATA
Foxo1-mut	ACCTTGTGACCACCTCTGAGTAATATAAGTC CTGCTA	ACTCAGAGGTGGTCACAAGGTCCACAAA CCTGCCTAGAGA
miR-802-chip	AGGTCTCCAGGGGACAAGGG	TAGCAGGACTTATATTACTCATCTT
wt-miR-802-EMSA	GGACCTTTGTCAACAAGATGAGTAA	TTACTCATCTTGTTGACAAAGGTCC
mut-miR-802 EMSA	GGACCTTGTGACCACCTCTGAGTAA	TTACTCAGAGGTGGTCACAAGGTCC

Supplementary Table 7A Oligo sequences used for sgRNA and shRNA

Gene	Forward strand (5'-3')	Reverse strand (5'-3')
miR-802-sgRNA-1	CACCGCCACATCACACTATCGAGAG	AAACCTCTCGATAGTGTGATGTGGC
miR-802-sgRNA-2	CACCGTCTCGATAGTGTGATGTGGC	AAACGCCACATCACACTATCGAGAC
miR-802-sgRNA-3	CACCGTAGAGAGTGCATCCTCGGAG	AAACCTCCGAGGATGCACTCTCTAC
miR-802-sgRNA-4	CACCGCACAGGAGCGCTCCACTCCG	AAACCGGAGTGGAGCGCTCCTGTGC
shCREB	GATCCGGGCAGTACATTGCCATTATTCAAG AGATAATGGCAATGTACTGCCC TTTTTTG	AATTCAAAAAAGGGCAGTACATTGCCATTAT CTCTTGAATAATGGCAATGTACTGCCCG

Supplementary Table 7B Target sequences used for siRNA

siRNA name	5'-3'
si-Fzd5	UCCUCUUAGUCUAUUUCUU
si-Neurod1	AUGCCUUUACCAUGCACUA
si-sox6	GUUCUGUCAUCUCAGCAAA
si-Foxo1	GAAUUCAAUUCGCCACAAU

Supplementary Table 8 Recombinant plasmids used in this paper

Name	Source	Application
PGL3-miR-802 promoter	This paper	Luciferase
PGL3-miR-802 R1	This paper	Luciferase
PGL3-Ins2 promoter	This paper	Luciferase
PGL3-Sox6 promoter	This paper	Luciferase
PGL3-Sox6 R1	This paper	Luciferase
PGL3-Sox6 R2	This paper	Luciferase
PGLO-Fzd5-WT	This paper	Luciferase
PGLO-Fzd5-Mut	This paper	Luciferase
PGLO-NeuroD1-WT	This paper	Luciferase
PGLO-NeuroD1-Mut	This paper	Luciferase
pcDNA 3.1-NeuroD1	This paper	Overexpression NeuroD1
pcDNA 3.1-Fzd5	This paper	Overexpression Fzd5
pcDNA 3.1-CREB	This paper	Overexpression CREB
pcDNA 3.1-DAD-Foxo1	This paper	Activation Foxo1
pcDNA 3.1-Sox6	This paper	Overexpression Sox6

Supplementary Table 9 Quantitative PCR primers sequences

Gene	Forward strand (5'-3')	Reverse strand (5'-3')
Mus-miR-802-5p	CGGCGTCAGTAACAAAGATTC	TATGGTTTTGACGACTGTGTGAT
Pri-mus-miR-802	TCCCCACCTGACTCTACATAACCT	CGTCCTCTCATCTTCCCTTTCGA
Hus-miR-802	GCCGCGCAGTAACAAAGATT	TATGGTTGTTCACGACTCCTTCAC
Foxo1	CCCAGGCCGGAGTTTAACC	GTTGCTCATAAAGTCGGTGCT
Fzd5	CCAAACCTACGCTCCCAGG	CGCACCTTGTTGTAGAGTGG
CREB	AGCAGCTCATGCAACATCATC	AGTCCTTACAGGAAGACTGAACT
Ins1	CACTTCCTACCCCTGCTGG	ACCACAAAGATGCTGTTTGACA
Ins2	GCTTCTTCTACACACCCATGTC	AGCACTGATCTACAATGCCAC
NeuroD1	ATGACCAAATCATACAGCGAGAG	TCTGCCTCGTGTTCCTCGT
Sox6	GGTCATGTTTCCCACCCACAA	TTCAGAGGGGTCCAAATTCCT
Hes1	CCAGCCAGTGTCAACACGA	AATGCCGGGAGCTATCTTTCT
Bhlhe22	TGAACGACGCTCTGGATGAG	GGTTGAGGTAGGCGACTAAGC
Pdx1	CCCCAGTTTACAAGCTCGCT	CTCGGTTCCATTCGGGAAAGG
MafA	AGGAGGAGGTCATCCGACTG	CTTCTCGCTCTCCAGAATGTG
Nkx6.1	CTGCACAGTATGGCCGAGATG	CCGGGTTATGTGAGCCCAA
Pax6	TACCAGTGTCTACCAGCCAAT	TGCACGAGTATGAGGAGGTCT
Nkx2.2	AAGCATTTCAAAACCGACGGA	CCTCAAATCCACAGATGACCAGA
Gapdh	AGGTCGGTGTGAACGGATTTG	TGTAGACCATGTAGTTGAGGTCA

Supplementary Table 10 The antibodies used in Western blot and immunofluorescence

Protein	Catalog	Dilution for Western blot	Dilution for IF
Foxo1	Abcam (ab39670)	1:1000	
NeuroD1	CST(4373)	1:1000	
Fzd5	Abcam (ab14475)	1:1000	
CamkⅡ	CST (4436)	1:1000	
P-CamkⅡ	CST (12716)	1:1000	
CREB	Abcam (ab31387)	1:1000	
P-CREB	Abcam (ab32096)	1:1000	
Sox6	Abcam (ab64946)	1:1000	
Lamin B1	Abcam (ab133741)	1:2000	
GAPDH	Abcam (ab181602)	1:2000	
Insulin	R&D (MAB1417)		1:500
Glucagon	Abcam (EP3070)		1:250

Supplementary Table 11 A the actual number of miR-802 KI mice (F0 to F1) for each genotype

Genetype	number	female	male
Rip-Cre miR-802$^{ki/wt}$	28	8	20
miR-802$^{ki/wt}$	24	7	17
Total	52	15	37

Supplementary Table 11 B the actual number of miR-802 KI mice (F1 to F2) for each genotype

Genetype	number	female	male
Rip-Cre miR-802$^{ki/ki}$	48	16	32
Rip-Cre miR-802$^{ki/wt}$	49	16	33
miR-802$^{ki/ki}$	46	15	31
miR-802$^{ki/wt}$	41	14	27
Total	184	61	123

Supplementary Table 12 A the actual number of miR-802 KO (F0 to F1) mice for each genotype

Genetype	number	female	male
Rip-Cre miR-802$^{fl/wt}$	39	12	27
miR-802$^{fl/wt}$	37	10	27
Total	76	24	52

Supplementary Table 12 B the actual number of miR-802 KO (F1 to F2) mice for each genotype

Genetype	number	female	male
Rip-Cre miR-802$^{fl/fl}$	73	21	52
Rip-Cre miR-802$^{fl/wt}$	74	24	50
miR-802$^{fl/fl}$	69	19	50
miR-802$^{fl/wt}$	68	21	47
Total	284	85	199
