# Supplementary material for: Obesity-induced overexpression of miR-802 impairs insulin transcription and secretion
Source: Nat Commun. 2020 Apr 14;11:1822. doi: 10.1038/s41467-020-15529-w (PMC7156651; doi:10.1038/s41467-020-15529-w)
Supplement: Supplementary file 3 — Reporting Summary [file 41467_2020_15529_MOESM3_ESM.pdf]

nature research

Corresponding author(s): Xianghui Fu, Zhengyu Cao, Liang Jin

Last updated by author(s): Feb 8, 2020

Reporting Summary

Nature Research wishes to improve the reproducibility of the work that we publish. This form provides structure for consistency and transparency in reporting. For further information on Nature Research policies, see Authors & Referees and the Editorial Policy Checklist .

Statistics

For all statistical analyses, confirm that the following items are present in the figure legend, table legend, main text, or Methods section.

n/a	Confirmed	
☐	☒	The exact sample size (*n*) for each experimental group/condition, given as a discrete number and unit of measurement
☐	☒	A statement on whether measurements were taken from distinct samples or whether the same sample was measured repeatedly
☐	☒	The statistical test(s) used AND whether they are one- or two-sided<br>*Only common tests should be described solely by name; describe more complex techniques in the Methods section.*
☐	☒	A description of all covariates tested
☐	☒	A description of any assumptions or corrections, such as tests of normality and adjustment for multiple comparisons
☐	☒	A full description of the statistical parameters including central tendency (e.g. means) or other basic estimates (e.g. regression coefficient) AND variation (e.g. standard deviation) or associated estimates of uncertainty (e.g. confidence intervals)
☐	☒	For null hypothesis testing, the test statistic (e.g. *F*, *t*, *r*) with confidence intervals, effect sizes, degrees of freedom and *P* value noted<br>*Give P values as exact values whenever suitable.*
☐	☒	For Bayesian analysis, information on the choice of priors and Markov chain Monte Carlo settings
☐	☒	For hierarchical and complex designs, identification of the appropriate level for tests and full reporting of outcomes
☐	☒	Estimates of effect sizes (e.g. Cohen's *d*, Pearson's *r*), indicating how they were calculated

Our web collection on statistics for biologists contains articles on many of the points above.

Software and code

Policy information about availability of computer code

Data collection	There were no software and special code used in our manuscript.
Data analysis	FACS data was analyzed using FlowJo v10. Images were processed by ImageJ. Differential expression of RNA-Seq data was measured by DESeq version 1.26.0. Granule number was analyzed via MetaMorph Software version 7.7 was used for analysing . Statistical data analysis and figures were generated GraphPad Prism v7.

For manuscripts utilizing custom algorithms or software that are central to the research but not yet described in published literature, software must be made available to editors/reviewers. We strongly encourage code deposition in a community repository (e.g. GitHub). See the Nature Research guidelines for submitting code & software for further information.

Data

Policy information about availability of data

All manuscripts must include a data availability statement. This statement should provide the following information, where applicable:

- Accession codes, unique identifiers, or web links for publicly available datasets
- A list of figures that have associated raw data
- A description of any restrictions on data availability

Source data are provided as a Source Data file, and the RNA-Sequence data were deposited in the NCBI's Sequence Read Archive (SRA) database (PRJNA577478, and PRJNA577480).

Field-specific reporting

Please select the one below that is the best fit for your research. If you are not sure, read the appropriate sections before making your selection.

Life sciences study design

All studies must disclose on these points even when the disclosure is negative.

Sample size	All experiments were performed in three independent experiments, each performed in triplicate in this study. Two-tailed unpaired Student's t test was used to show the significant difference between 2 groups, and ANOVA was used for multigroup difference analysis. Dunn's multiple comparisons for one-way ANOVA and Fisher's least signicant difference (LSD) for two-way ANOVA were used.
Data exclusions	No data were excluded from the analyses in this study.
Replication	Reported results were tested and confirmed in cell lines. Reported results considered biological replicates (in vivo n=6 and in vitro n=3 in technical duplicates). All attempts at replication were successful and all details on biological and technical replicates are provided in the text and/or figure legends.
Randomization	Allocation of all animal experiments were random. All animals in these studies were maintained under the same conditions.
Blinding	For In-vitro experiments, investigators were blinded to group allocation during data collection/analysis.

Behavioural & social sciences study design

All studies must disclose on these points even when the disclosure is negative.

Study description	*Briefly describe the study type including whether data are quantitative, qualitative, or mixed-methods (e.g. qualitative cross-sectional, quantitative experimental, mixed-methods case study).*
Research sample	*State the research sample (e.g. Harvard university undergraduates, villagers in rural India) and provide relevant demographic information (e.g. age, sex) and indicate whether the sample is representative. Provide a rationale for the study sample chosen. For studies involving existing datasets, please describe the dataset and source.*
Sampling strategy	*Describe the sampling procedure (e.g. random, snowball, stratified, convenience). Describe the statistical methods that were used to predetermine sample size OR if no sample-size calculation was performed, describe how sample sizes were chosen and provide a rationale for why these sample sizes are sufficient. For qualitative data, please indicate whether data saturation was considered, and what criteria were used to decide that no further sampling was needed.*
Data collection	*Provide details about the data collection procedure, including the instruments or devices used to record the data (e.g. pen and paper, computer, eye tracker, video or audio equipment) whether anyone was present besides the participant(s) and the researcher, and whether the researcher was blind to experimental condition and/or the study hypothesis during data collection.*
Timing	*Indicate the start and stop dates of data collection. If there is a gap between collection periods, state the dates for each sample cohort.*
Data exclusions	*If no data were excluded from the analyses, state so OR if data were excluded, provide the exact number of exclusions and the rationale behind them, indicating whether exclusion criteria were pre-established.*
Non-participation	*State how many participants dropped out/declined participation and the reason(s) given OR provide response rate OR state that no participants dropped out/declined participation.*
Randomization	*If participants were not allocated into experimental groups, state so OR describe how participants were allocated to groups, and if allocation was not random, describe how covariates were controlled.*

Ecological, evolutionary & environmental sciences study design

All studies must disclose on these points even when the disclosure is negative.

Study description	*Briefly describe the study. For quantitative data include treatment factors and interactions, design structure (e.g. factorial, nested, hierarchical), nature and number of experimental units and replicates.*
Research sample	*Describe the research sample (e.g. a group of tagged Passer domesticus, all Stenocereus thurberi within Organ Pipe Cactus National Monument), and provide a rationale for the sample choice. When relevant, describe the organism taxa, source, sex, age range and any manipulations. State what population the sample is meant to represent when applicable. For studies involving existing datasets, describe the data and its source.*
Sampling strategy	*Note the sampling procedure. Describe the statistical methods that were used to predetermine sample size OR if no sample-size calculation was performed, describe how sample sizes were chosen and provide a rationale for why these sample sizes are sufficient.*
Data collection	*Describe the data collection procedure, including who recorded the data and how.*

Timing and spatial scale	*Indicate the start and stop dates of data collection, noting the frequency and periodicity of sampling and providing a rationale for these choices. If there is a gap between collection periods, state the dates for each sample cohort. Specify the spatial scale from which the data are taken*
Data exclusions	*If no data were excluded from the analyses, state so OR if data were excluded, describe the exclusions and the rationale behind them, indicating whether exclusion criteria were pre-established.*
Reproducibility	*Describe the measures taken to verify the reproducibility of experimental findings. For each experiment, note whether any attempts to repeat the experiment failed OR state that all attempts to repeat the experiment were successful.*
Randomization	*Describe how samples/organisms/participants were allocated into groups. If allocation was not random, describe how covariates were controlled. If this is not relevant to your study, explain why.*
Blinding	*Describe the extent of blinding used during data acquisition and analysis. If blinding was not possible, describe why OR explain why blinding was not relevant to your study.*

Did the study involve field work? ☐ Yes ☐ No

Field work, collection and transport

Field conditions	*Describe the study conditions for field work, providing relevant parameters (e.g. temperature, rainfall).*
Location	*State the location of the sampling or experiment, providing relevant parameters (e.g. latitude and longitude, elevation, water depth).*
Access and import/export	*Describe the efforts you have made to access habitats and to collect and import/export your samples in a responsible manner and in compliance with local, national and international laws, noting any permits that were obtained (give the name of the issuing authority, the date of issue, and any identifying information).*
Disturbance	*Describe any disturbance caused by the study and how it was minimized.*

Reporting for specific materials, systems and methods

We require information from authors about some types of materials, experimental systems and methods used in many studies. Here, indicate whether each material, system or method listed is relevant to your study. If you are not sure if a list item applies to your research, read the appropriate section before selecting a response.

Materials & experimental systems

n/a	Involved in the study	
☐	☒	Antibodies
☐	☒	Eukaryotic cell lines
☒	☐	Palaeontology
☐	☒	Animals and other organisms
☐	☒	Human research participants
☒	☐	Clinical data

Methods

n/a	Involved in the study	
☒	☐	ChIP-seq
☐	☒	Flow cytometry
☒	☐	MRI-based neuroimaging

Antibodies

Antibodies used	Foxo1 Abcam (ab39670) 1:1000 NeuroD1 CST(4373) 1:1000 Fzd5 Abcam (ab14475) 1:1000 Camk◦ CST (4436) 1:1000 P-Camk◦ CST (12716) 1:1000 CREB Abcam (ab31387) 1:1000 P-CREB Abcam (ab32096) 1:1000 Sox6 Abcam (ab64946) 1:1000 Lamin B1 Abcam (ab133741) 1:2000 GAPDH Abcam (ab181602) 1:2000 Insulin R&D (MAB1417) 1:500 Glucagon Abcam (EP3070) 1:250
Validation	Foxo1 Abcam (ab39670) 1:1000, Species: Rabbit, application: ICC/IF, WB, IHC-P, ELISA, ChIP<br>NeuroD1 CST(4373) 1:1000 , Species: Rabbit, application: WB, IP, ChIP<br>Fzd5 Abcam (ab14475) 1:1000, Species: Rabbit, application: ICC/IF, IHC-FoFr, WB<br>Camk◦ CST (4436) 1:1000, Species: Rabbit, application: WB<br>P-Camk◦ CST (12716) 1:1000, Species: Rabbit, application: WB<br>CREB Abcam (ab31387) 1:1000, Species: Rabbit, application: ChIP, ELISA, ICC/IF, IHC-P, WB<br>P-CREB Abcam (ab32096) 1:1000, Species: Rabbit, application: WB, IHC-P, IP, Flow Cyt, Dot blot, ICC/IF<br>Sox6 Abcam (ab64946) 1:1000, Species: Rabbit, application: ChIP/Chip, WB, ELISA, ICC/IF<br>Lamin B1 Abcam (ab133741) 1:2000, Species: Rabbit, application: IP, ICC/IF, WB, IHC-P<br>GAPDH Abcam (ab181602) 1:2000 , Species: Rabbit, application: WB, IHC-P, ICC/IF, Flow Cyt, IP<br>Insulin R&D (MAB1417) 1:500, Species: Human, Mouse, Bovine, application: ICC/IF

	Glucagon Abcam (EP3070) 1:250, Species: Rabbit, application: WB, IHC-P, IHC-Fr, ELISA

Eukaryotic cell lines

Policy information about cell lines

Cell line source(s)	Min6 cell was donated by Defu Zeng, Professor, from Departments of Diabetes Immunology and Hematopoietic Cell Transplantation Irell & Manella Graduate School of Biological Sciences of City of Hope which was not commercial.
Authentication	Primary islet cells were isolated from mouse pancreas by collagenase digestion followed by Histopaque density gradient. Primary islet cells were stained dithizone to authenticate<br>The mice pancreatic β-cell line Min6 was donated by Defu Zeng professor, Min6 cells were authenticated by qRT-PCR, IF and ELISA assays. qRT-PCR and IF were used to test insulin gene expression, and ELISA assays was used to quantify insulin secretion.
Mycoplasma contamination	cell lines were not tested for mycoplasma contaminatin.
Commonly misidentified lines (See ICLAC register)	Min6 cells, the mice pancreatic β-cell line, can synthesis insulin and secrete insulin. Thus, we choose Min6 cells to mimic the primary islet cells.

Palaeontology

Specimen provenance	*Provide provenance information for specimens and describe permits that were obtained for the work (including the name of the issuing authority, the date of issue, and any identifying information).*
Specimen deposition	*Indicate where the specimens have been deposited to permit free access by other researchers.*
Dating methods	*If new dates are provided, describe how they were obtained (e.g. collection, storage, sample pretreatment and measurement), where they were obtained (i.e. lab name), the calibration program and the protocol for quality assurance OR state that no new dates are provided.*

☐ Tick this box to confirm that the raw and calibrated dates are available in the paper or in Supplementary Information.

Animals and other organisms

Policy information about studies involving animals; ARRIVE guidelines recommended for reporting animal research

Laboratory animals	8 weeks C57BL/6J male mice and db/db male mice (8 weeks), all animals were on the C57BL/6 background except db/db mice, which were on the BKS background (Janvier). C57BL/6J mice were fed High Fat Diet (HFD) for 8 weeks (D12494, 60% energy from fat) according to the criteria defined by Peyot ML , weighted between 40 and 45 g. The control groups were fed with normal diet (D12450J, 10% energy from fat), weighted between 23 and 25 g.
Wild animals	No wild animals were used in the study
Field-collected samples	No field-collected samples were used in the study.
Ethics oversight	All care and handling of animals were carried out according to the international laws and policies (EEC Council Directive 86/609, 1987) and approved by the animal ethics committee of China Pharmaceutical University (Nanjing, China) Care of animals was within institutional animal-care committee guidelines.

Note that full information on the approval of the study protocol must also be provided in the manuscript.

Human research participants

Policy information about studies involving human research participants

Population characteristics	lean individuals (20 ≤ BMI ≤ 25), age between(23-71); and obesity (BMI > 25), age between(26-67), 52 male and 45 female.
Recruitment	Individuals, satisfied the clinical features (lean individuals (20 ≤ BMI ≤ 25) or obesity (BMI > 25), were randomly recruited. Almost no any potential self-selection bias or other biases that may be present and impact results.
Ethics oversight	The experiments were approved by the Ethics Committees of the Department of Zhongda Hospital Southeast University (Nanjing, China).

Note that full information on the approval of the study protocol must also be provided in the manuscript.

Clinical data

Policy information about clinical studies

All manuscripts should comply with the ICMJEguidelines for publication of clinical research and a completedCONSORT checklist must be included with all submissions.

Clinical trial registration	*Provide the trial registration number from ClinicalTrials.gov or an equivalent agency.*
Study protocol	*Note where the full trial protocol can be accessed OR if not available, explain why.*
Data collection	*Describe the settings and locales of data collection, noting the time periods of recruitment and data collection.*
Outcomes	*Describe how you pre-defined primary and secondary outcome measures and how you assessed these measures.*

ChIP-seq

Data deposition

☐ Confirm that both raw and final processed data have been deposited in a public database such as GEO.

☐ Confirm that you have deposited or provided access to graph files (e.g. BED files) for the called peaks.

Data access links<br>*May remain private before publication.*	*For "Initial submission" or "Revised version" documents, provide reviewer access links. For your "Final submission" document, provide a link to the deposited data.*
Files in database submission	*Provide a list of all files available in the database submission.*
Genome browser session<br>(e.g. UCSC)	*Provide a link to an anonymized genome browser session for "Initial submission" and "Revised version" documents only, to enable peer review. Write "no longer applicable" for "Final submission" documents.*

Methodology

Replicates	*Describe the experimental replicates, specifying number, type and replicate agreement.*
Sequencing depth	*Describe the sequencing depth for each experiment, providing the total number of reads, uniquely mapped reads, length of reads and whether they were paired- or single-end.*
Antibodies	*Describe the antibodies used for the ChIP-seq experiments; as applicable, provide supplier name, catalog number, clone name, and lot number.*
Peak calling parameters	*Specify the command line program and parameters used for read mapping and peak calling, including the ChIP, control and index files used.*
Data quality	*Describe the methods used to ensure data quality in full detail, including how many peaks are at FDR 5% and above 5-fold enrichment.*
Software	*Describe the software used to collect and analyze the ChIP-seq data. For custom code that has been deposited into a community repository, provide accession details.*

Flow Cytometry

Plots

Confirm that:

☒ The axis labels state the marker and fluorochrome used (e.g. CD4-FITC).

☒ The axis scales are clearly visible. Include numbers along axes only for bottom left plot of group (a 'group' is an analysis of identical markers).

☒ All plots are contour plots with outliers or pseudocolor plots.

☒ A numerical value for number of cells or percentage (with statistics) is provided.

Methodology

Sample preparation	After 48 h transfection, cells were treated with trypsin without Ethylenediamine tetraacetic acid (EDTA), and according to the instructions of Annexin V-FITC Apoptosis detection kit (KeyGEN BioTECH, Nanjing, Jiangsu, china). The FITC and PI were tested at 488 nm and 630 nm for the detection of cell apoptosis.
Instrument	State Key Laboratory of Natural Medicines, Jiangsu Key Laboratory of Druggability of Biopharmaceuticals, School of life Science and Technology, China Pharmaceutical University.

Software	FlowJo 10 and BD Accuri C6 software.
Cell population abundance	about 2x10^6 cells.
Gating strategy	The preliminary pi/FITC gates of the sorting cell population . Apoptosis cells were both positive in PI and FITC .

☒ Tick this box to confirm that a figure exemplifying the gating strategy is provided in the Supplementary Information.

Magnetic resonance imaging

Experimental design

Design type	*Indicate task or resting state; event-related or block design.*
Design specifications	*Specify the number of blocks, trials or experimental units per session and/or subject, and specify the length of each trial or block (if trials are blocked) and interval between trials.*
Behavioral performance measures	*State number and/or type of variables recorded (e.g. correct button press, response time) and what statistics were used to establish that the subjects were performing the task as expected (e.g. mean, range, and/or standard deviation across subjects).*

Acquisition

Imaging type(s)	*Specify: functional, structural, diffusion, perfusion.*
Field strength	*Specify in Tesla*
Sequence & imaging parameters	*Specify the pulse sequence type (gradient echo, spin echo, etc.), imaging type (EPI, spiral, etc.), field of view, matrix size, slice thickness, orientation and TE/TR/flip angle.*
Area of acquisition	*State whether a whole brain scan was used OR define the area of acquisition, describing how the region was determined.*

Diffusion MRI ☐ Used ☐ Not used

Preprocessing

Preprocessing software	*Provide detail on software version and revision number and on specific parameters (model/functions, brain extraction, segmentation, smoothing kernel size, etc.).*
Normalization	*If data were normalized/standardized, describe the approach(es): specify linear or non-linear and define image types used for transformation OR indicate that data were not normalized and explain rationale for lack of normalization.*
Normalization template	*Describe the template used for normalization/transformation, specifying subject space or group standardized space (e.g. original Talairach, MNI305, ICBM152) OR indicate that the data were not normalized.*
Noise and artifact removal	*Describe your procedure(s) for artifact and structured noise removal, specifying motion parameters, tissue signals and physiological signals (heart rate, respiration).*
Volume censoring	*Define your software and/or method and criteria for volume censoring, and state the extent of such censoring.*

Statistical modeling & inference

Model type and settings	*Specify type (mass univariate, multivariate, RSA, predictive, etc.) and describe essential details of the model at the first and second levels (e.g. fixed, random or mixed effects; drift or auto-correlation).*
Effect(s) tested	*Define precise effect in terms of the task or stimulus conditions instead of psychological concepts and indicate whether ANOVA or factorial designs were used.*

Specify type of analysis: ☐ Whole brain ☐ ROI-based ☐ Both

Statistic type for inference (See Eklund et al. 2016)	*Specify voxel-wise or cluster-wise and report all relevant parameters for cluster-wise methods.*
Correction	*Describe the type of correction and how it is obtained for multiple comparisons (e.g. FWE, FDR, permutation or Monte Carlo).*

Models & analysis

n/a	Involved in the study
☐	☐ Functional and/or effective connectivity
☐	☐ Graph analysis
☐	☐ Multivariate modeling or predictive analysis

Functional and/or effective connectivity	*Report the measures of dependence used and the model details (e.g. Pearson correlation, partial correlation, mutual information).*
Graph analysis	*Report the dependent variable and connectivity measure, specifying weighted graph or binarized graph, subject- or group-level, and the global and/or node summaries used (e.g. clustering coefficient, efficiency, etc.).*
Multivariate modeling and predictive analysis	*Specify independent variables, features extraction and dimension reduction, model, training and evaluation metrics.*
