# Supplementary material for: Obesity-induced overexpression of miR-802 impairs insulin transcription and secretion
Source: Nat Commun. 2020 Apr 14;11:1822. doi: 10.1038/s41467-020-15529-w (PMC7156651; doi:10.1038/s41467-020-15529-w)

Fig.2 c

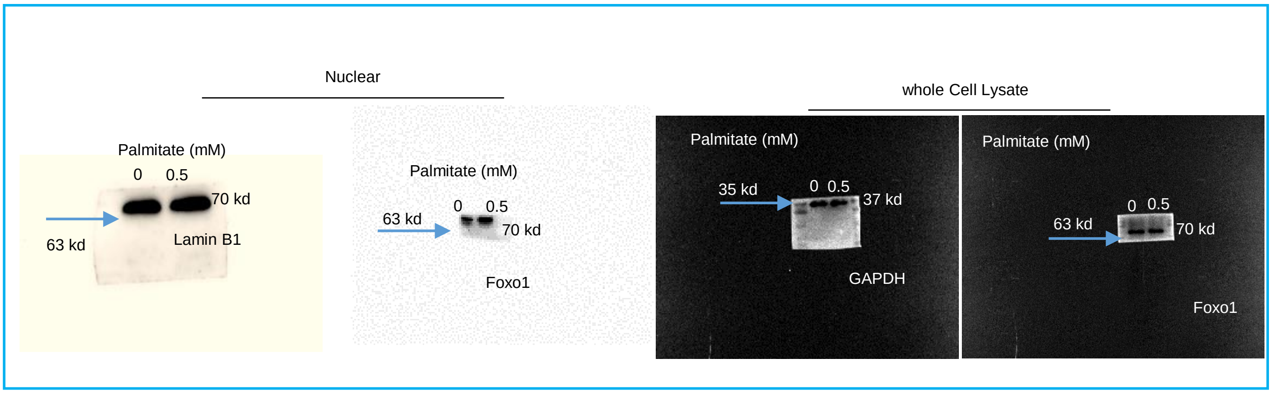

Fig.2 d

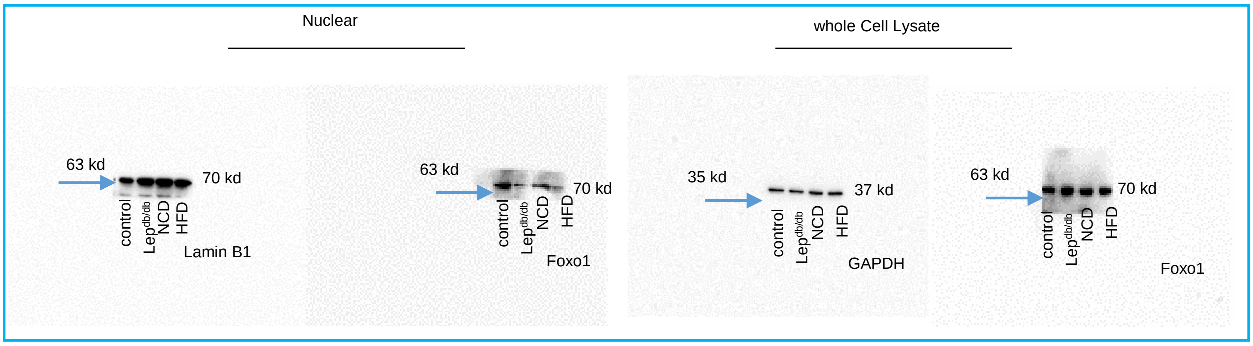

Fig.2h

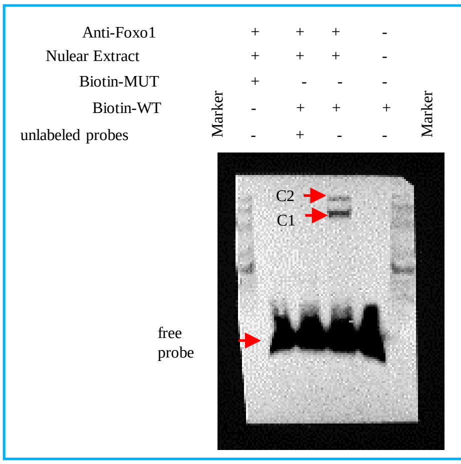

Fig.2i

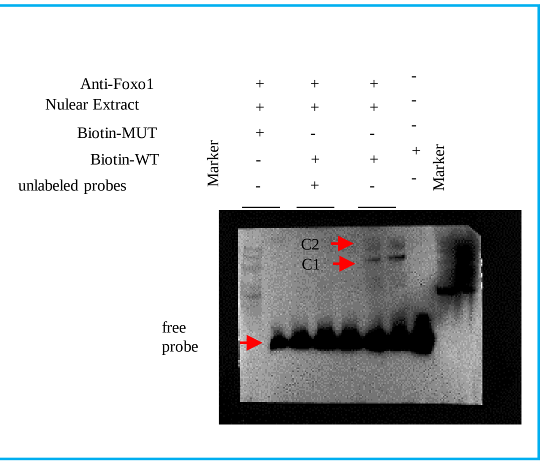

Fig.6a

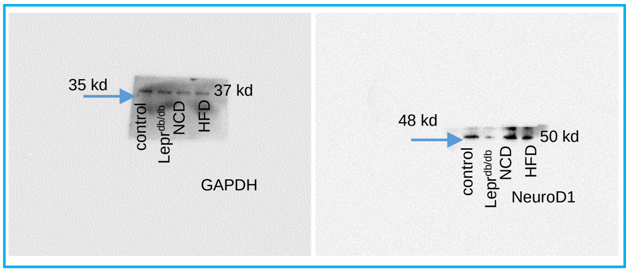

Fig.6b

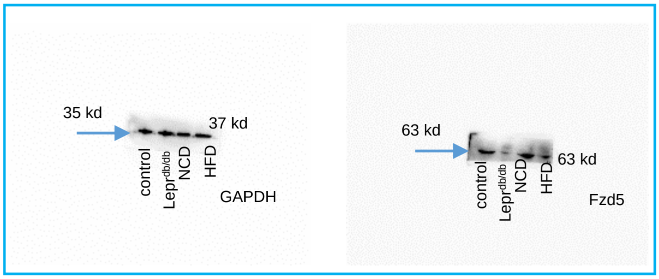

Fig.6c

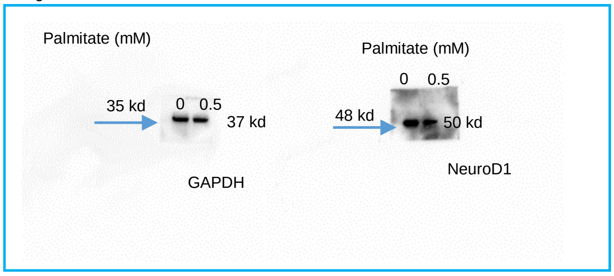

Fig.6d

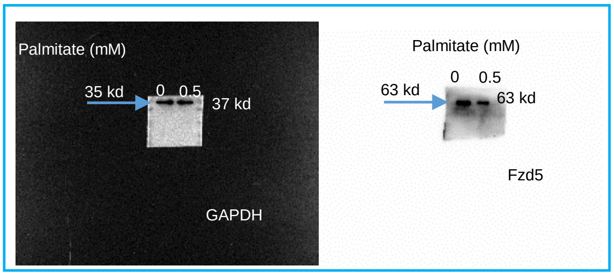

Fig.6g

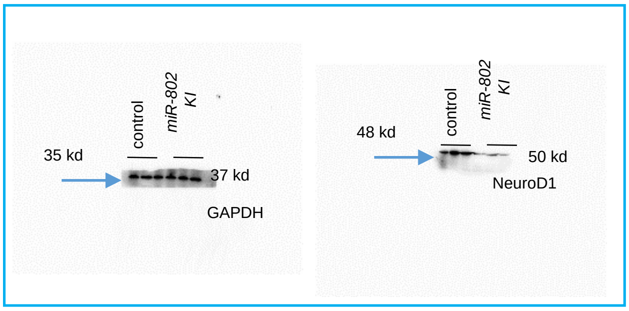

Fig.6h

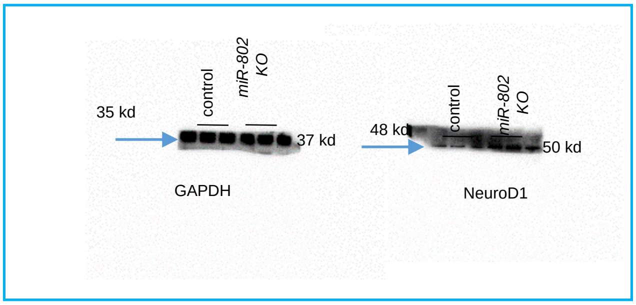

Fig.6i

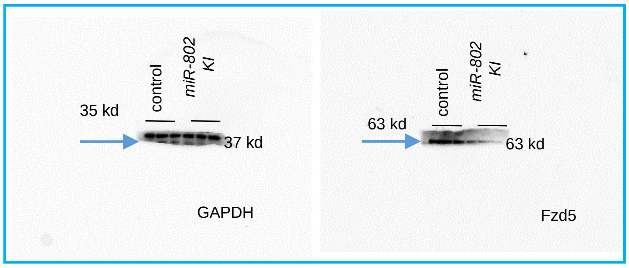

Fig.6j

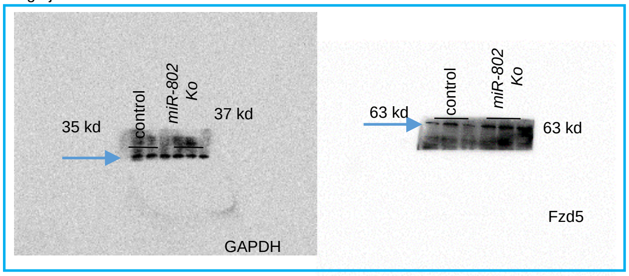

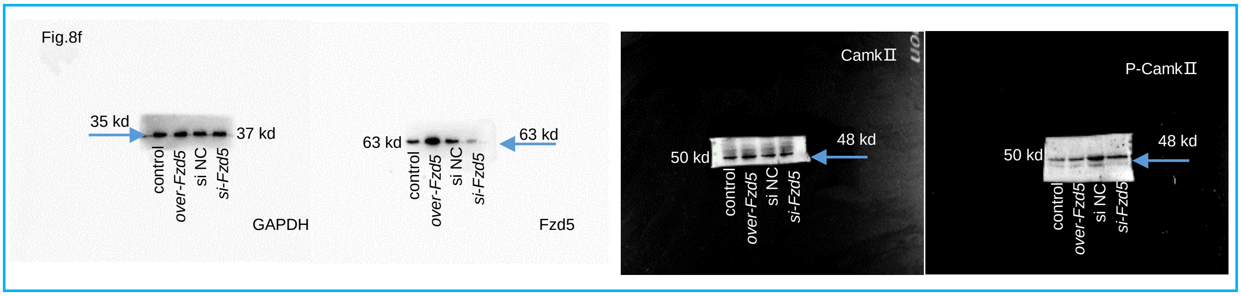

Fig.8j

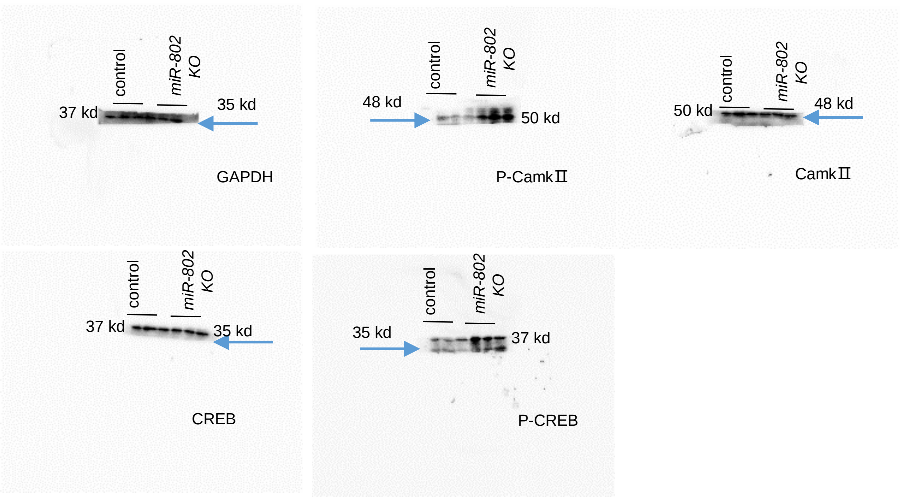

Fig.8k

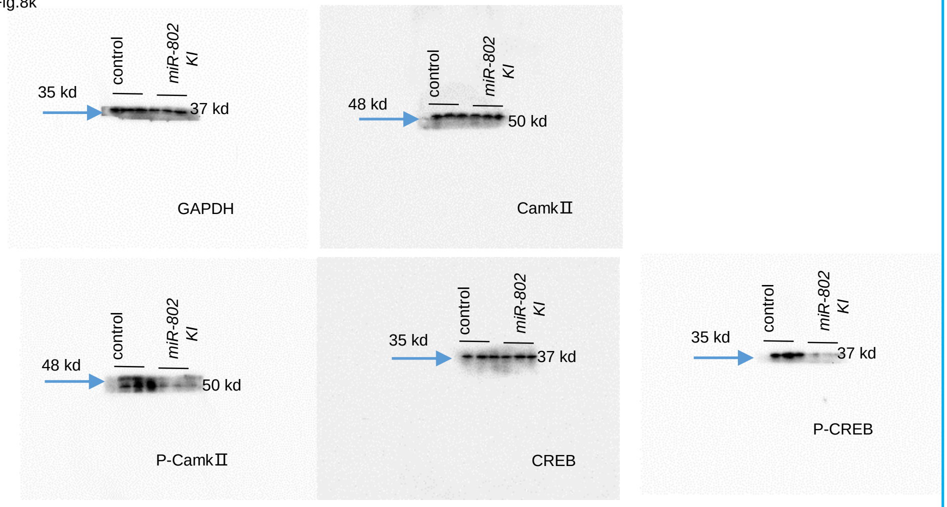

Fig.8l

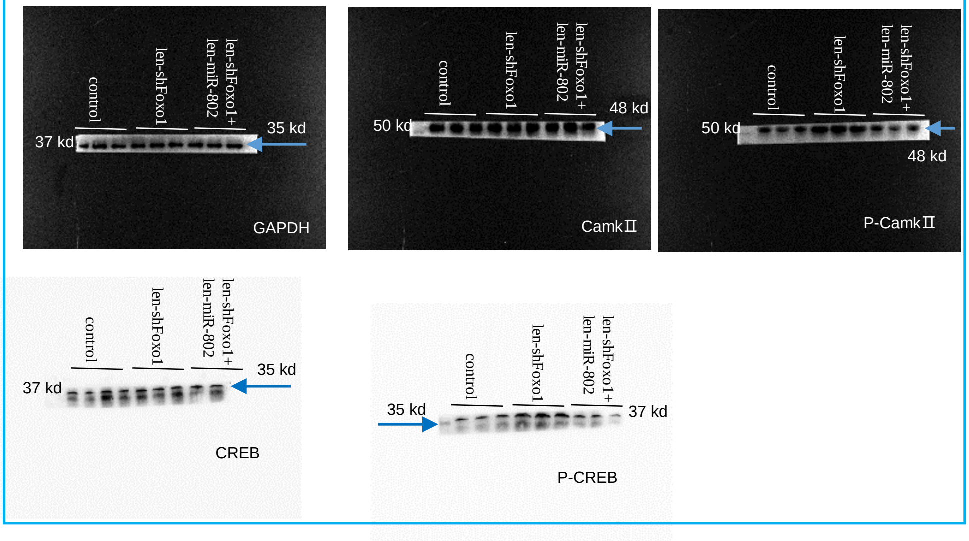

Fig.8m

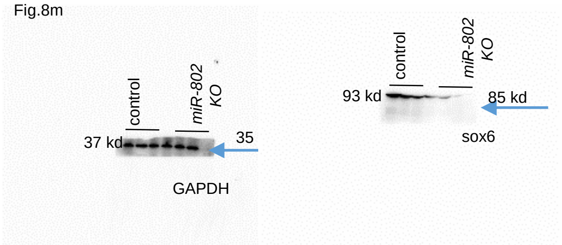

Fig.8n

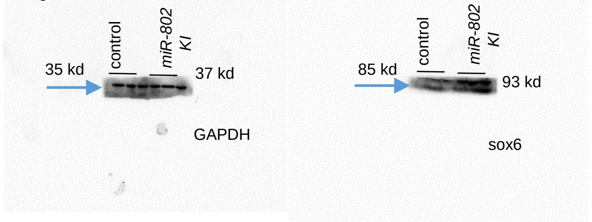

Supplementary Fig.2g

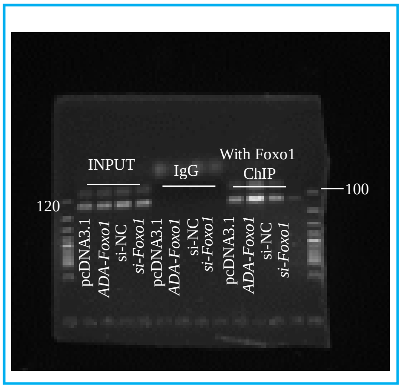

Supplementary Fig.2h

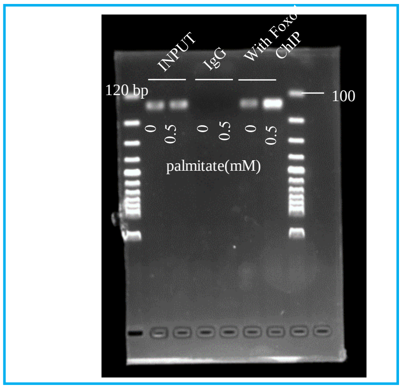

Supplementary Fig.2i

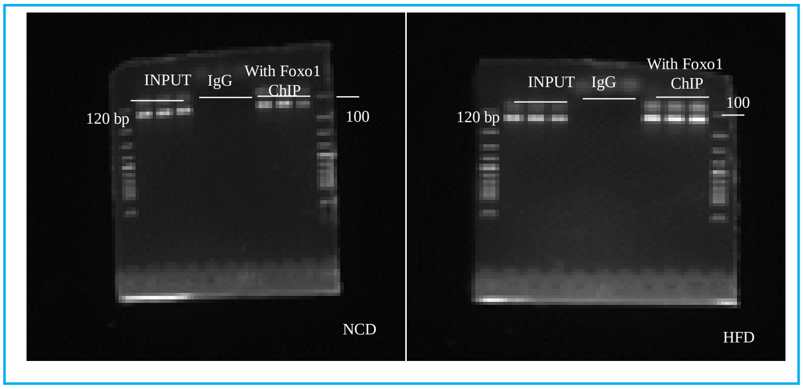

Supplementary Fig.2k

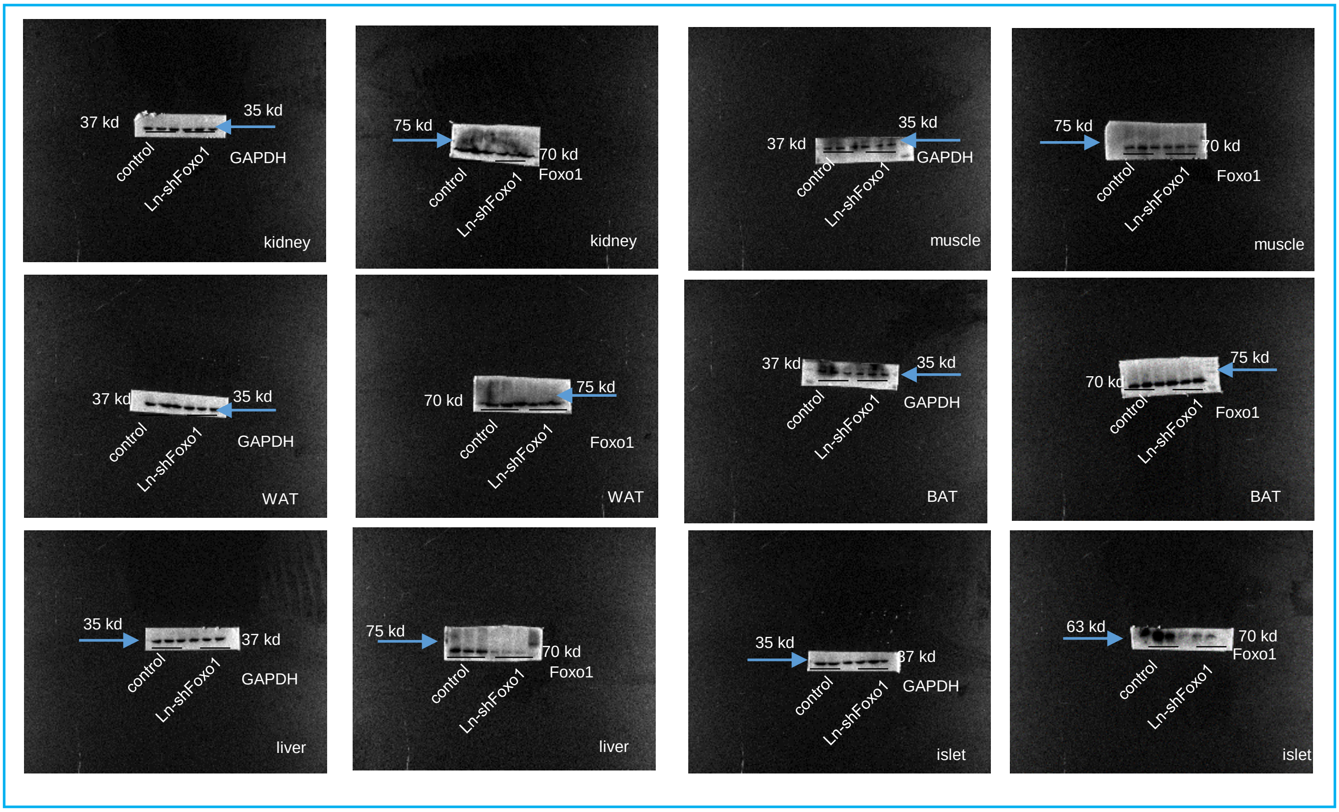

Supplementary Fig.3f

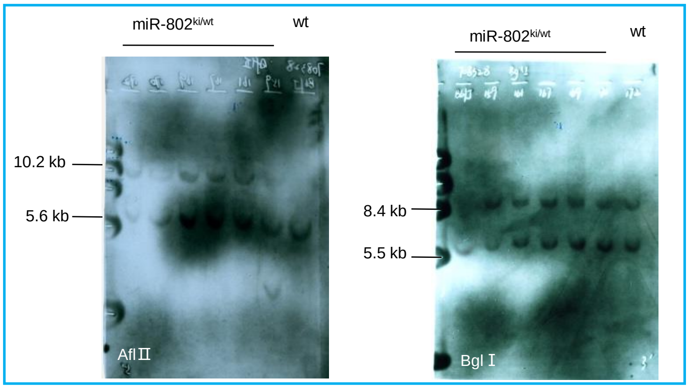

Supplementary Fig.3h

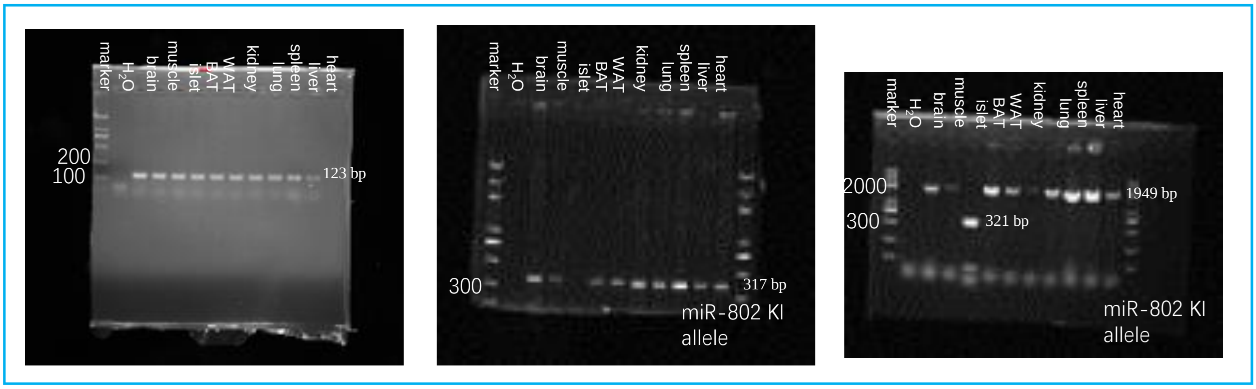

Supplementary Fig.3j

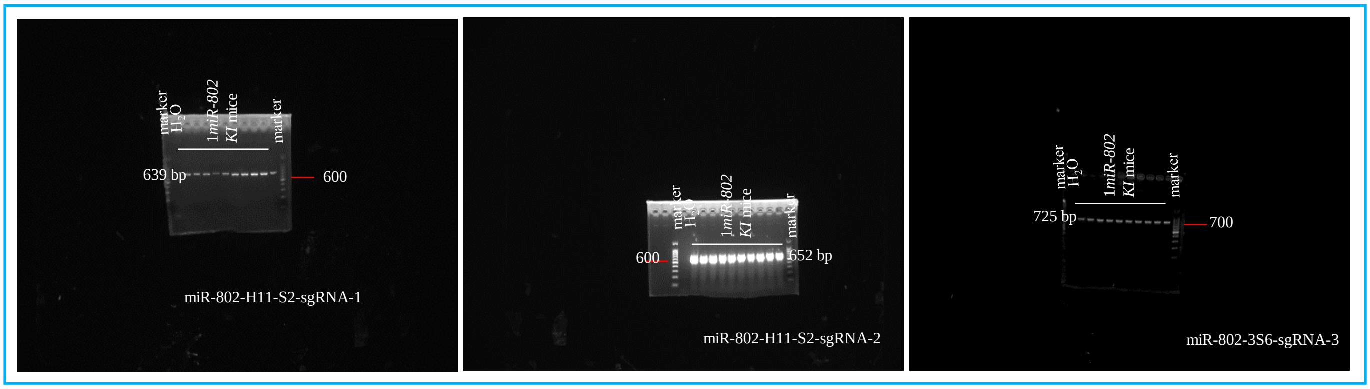

Supplementary Fig.4h

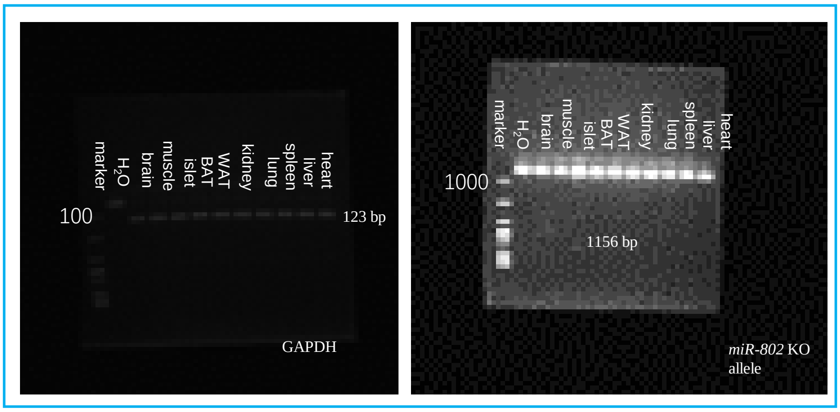

Supplementary Fig.4f

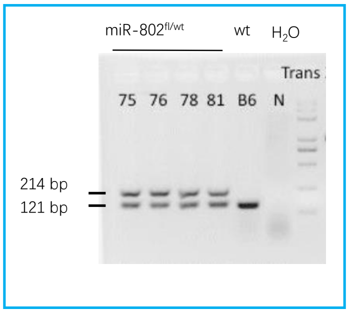

Supplementary Fig.4j

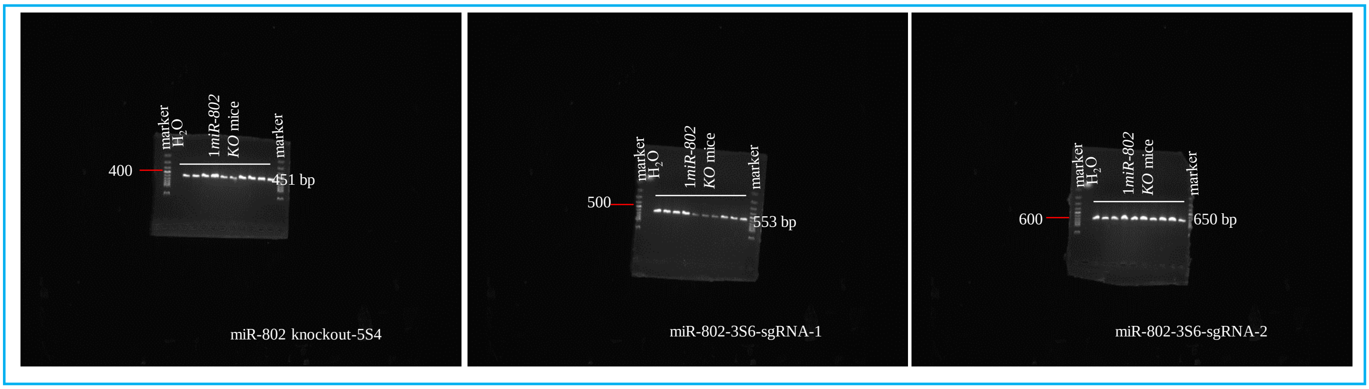

Supplementary Fig.6d

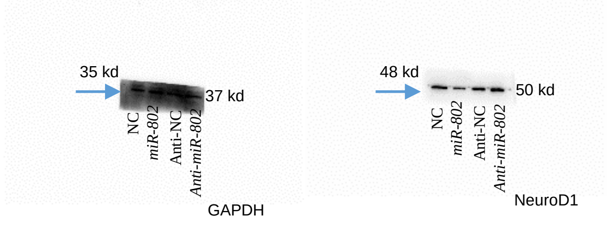

Supplementary Fig.6e

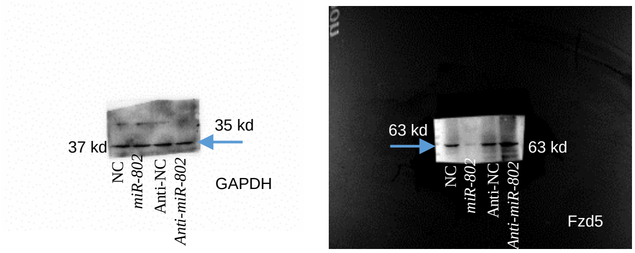

Supplementary Fig.6h

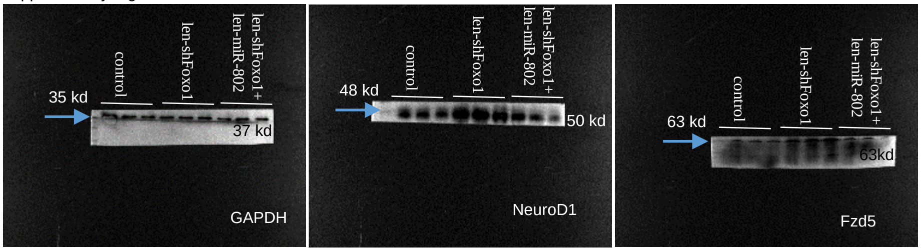

Supplementary Fig.8e

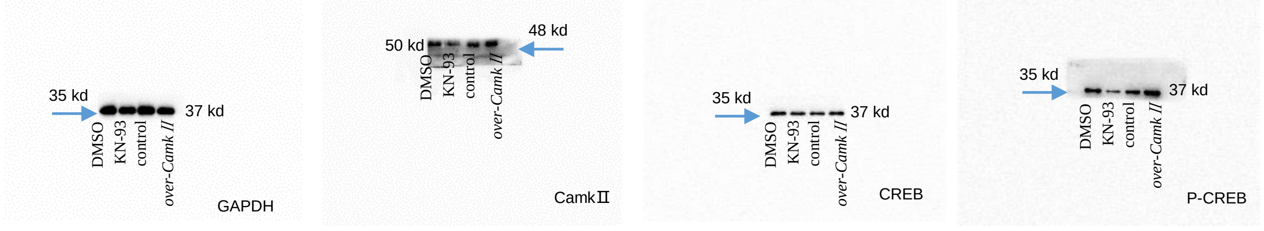

Supplementary Fig.8g

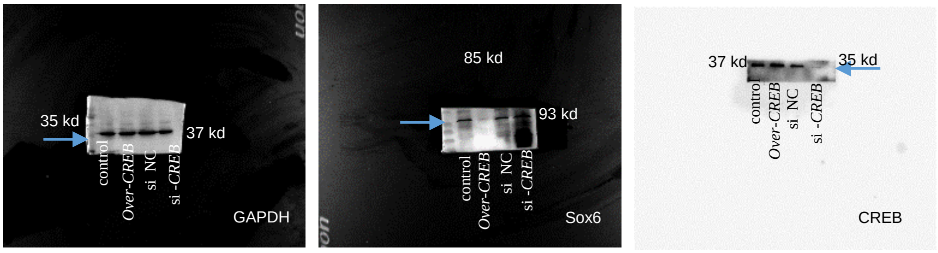

Supplementary Fig.8i

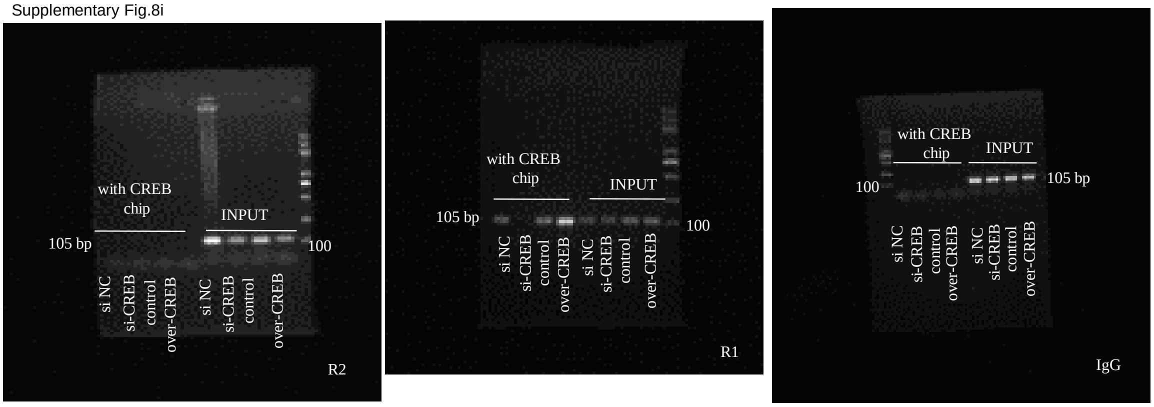

Supplement: Supplementary file 4 — Source Data [file 41467_2020_15529_MOESM4_ESM.zip › source data/source data of gels.pdf]
